# Genome-Wide Identification and Characterization of *R2R3-MYB* Provide Insight into Anthocyanin Biosynthesis Regulation Mechanism of *Ananas comosus* var. *bracteatus*

**DOI:** 10.3390/ijms24043133

**Published:** 2023-02-05

**Authors:** Wei Yang, Lijun Feng, Jiaheng Luo, Huiling Zhang, Fuxing Jiang, Yehua He, Xi Li, Juan Du, Mark Owusu Adjei, Aiping Luan, Jun Ma

**Affiliations:** 1 College of Landscape Architecture, Sichuan Agricultural University, Chengdu 625014, China; 2 College of Horticulture, South China Agricultural University, Guangzhou 510642, China; 3 Key Laboratory of Tropical Crops Germplasm Resources Genetic Improvement and Innovation of Hainan Province, Tropical Crops Genetic Resources Institute, Chinese Academy of Tropical Agricultural Sciences, Haikou 571101, China

**Keywords:** *Ananas comosus* var. *bracteatus*, genome-wide, R2R3-MYB, anthocyanin biosynthesis

## Abstract

The R2R3-MYB proteins comprise the largest class of MYB transcription factors, which play an essential role in regulating anthocyanin synthesis in various plant species. *Ananas comosus* var. *bracteatus* is an important colorful anthocyanins-rich garden plant. The spatio-temporal accumulation of anthocyanins in chimeric leaves, bracts, flowers, and peels makes it an important plant with a long ornamental period and highly improves its commercial value. We conducted a comprehensive bioinformatic analysis of the *R2R3-MYB* gene family based on genome data from *A. comosus* var. *bracteatus*. Phylogenetic analysis, gene structure and motif analysis, gene duplication, collinearity, and promoter analysis were used to analyze the characteristics of this gene family. In this work, a total of 99 *R2R3-MYB* genes were identified and classified into 33 subfamilies according to phylogenetic analysis, and most of them were localized in the nucleus. We found these genes were mapped to 25 chromosomes. Gene structure and protein motifs were conserved among *AbR2R3-MYB* genes, especially within the same subfamily. Collinearity analysis revealed four pairs of tandem duplicated genes and 32 segmental duplicates in *AbR2R3-MYB* genes, indicating that segmental duplication contributed to the amplification of the *AbR2R3-MYB* gene family. A total of 273 ABRE responsiveness, 66 TCA elements, 97 CGTCA motifs, and TGACG motifs were the main cis elements in the promoter region under response to ABA, SA, and MEJA. These results revealed the potential function of *AbR2R3-MYB* genes in response to hormone stress. Ten R2R3-MYBs were found to have high homology to MYB proteins reported to be involved in anthocyanin biosynthesis from other plants. RT-qPCR results revealed the 10 *AbR2R3-MYB* genes showed tissue-specific expression patterns, six of them expressed the highest in the flower, two genes in the bract, and two genes in the leaf. These results suggested that these genes may be the candidates that regulate anthocyanin biosynthesis of *A*. *comosus* var. *bracteatus* in the flower, leaf, and bract, respectively. In addition, the expressions of these 10 *AbR2R3-MYB* genes were differentially induced by ABA, MEJA, and SA, implying that these genes may play crucial roles in hormone-induced anthocyanin biosynthesis. Our study provided a comprehensive and systematic analysis of *AbR2R3-MYB* genes and identified the *AbR2R3-MYB* genes regulating the spatial-temporal anthocyanin biosynthesis in *A. comosus* var. *bracteatus*, which would be valuable for further study on the anthocyanin regulation mechanism of *A. comosus* var. *bracteatus.*

## 1. Introduction

The MYB superfamily is one of the largest transcription factor (TF) families with diverse functions in all eukaryotes. All the members have a highly conserved MYB domain located at the N-terminus [1] This domain generally consists of up to four imperfect amino acid sequence repeats (R) of about 52 amino acids, each forming three α-helices. The second and third helices form a helix-turn-helix (HTH) structure containing three regularly spaced tryptophan (or hydrophobic) residues, and these residues act as a hydrophobic core and are of great significance for maintaining the configuration of the HTH structure [2]. According to the number of MYB repeats, MYB proteins generally can be split into four classes (1R-MYB, R2R3-MYB, 3R-MYB, 4R-MYB) [3]. Among these four MYB classes, R2R3-MYB proteins are the most numerous type of protein in plants with two repeats at the N-terminus, and typically have transcriptional activation functions at the C-terminus [4]. In general, they are extensively involved in cell differentiation, hormone response, secondary metabolism, environmental stress, as well as resistance to diseases and insects [1,5].

Anthocyanins are multifunctional, water-soluble compounds. In plants, anthocyanins provide colorations in the flowers, fruits, stems, and leaves of plants ranging from red to purple. Many studies have identified that the anthocyanin metabolism pathway is conserved in higher plants [6,7,8]. Enzyme-coding structural genes related to this pathway, such as *phenylalanine ammonia-lyase* (*PAL*), *Chalcone synthase* (*CHS*), and *Chalcone isomerase* (*CHI*), have been widely characterized in many plants. In addition, their temporal and spatial expression has also been shown to be regulated at transcription by TFs [9]. MYB TFs have been confirmed to be the predominant regulator in anthocyanin biosynthesis. They generally control the anthocyanin level by self-regulating or forming an MBW (MYB-bHLH-WD40) complex with basic-helix-loop-helix (bHLH) and WD40 regulators to regulate the expression of downstream target genes [10,11].

Being one of the largest classes of MYB group in higher plants, R2R3-MYBs play an important role in regulating anthocyanin metabolism in horticultural plants. R2R3-MYBs have been reported to promote or suppress the expression of the structural genes of the anthocyanin biosynthesis pathway that determine the colors of plant tissues or organs. PhAN2 played an important role in the red color formation of the corolla in petunias [12,13]. MYB1 and WDR1 interacted coordinately with bHLH2 to promote the structural genes for anthocyanin metabolism in the flower limbs and tubes of the morning glory [14]. In *Lilium* spp., LhMYB6 and LhMYB12, a homolog of petunia AN2, determined organ- and tissue-specific accumulation of anthocyanin by interacting with LhbHLH2 [15]. Chiou suggested that OgMYB1 (a homolog of PeMYB11) enhanced the formation of red pigments in lip tissues via the promotion of *OgCHI* and *OgDFR* transcription [16]. Similarly, NnMYB5 from *N. nucifera*, which is a homolog of EsAN2, induced anthocyanin in flowers [17]. In *Arabidopsis*, it has been shown to promote anthocyanin accumulation in seeds and flower stalks by up-regulating *TT1* [18]. In addition, R2R3-MYBs are widely studied in fruits and vegetables. The transcription level of *MrMYB1* was responsible for anthocyanin content and highly related to the diverse ripe fruit colors of Chinese bayberry (*Myrica rubra*), such as white, red, and dark red-purple [19]. In apricots, light-induced expression of *PaMYB10* stimulated anthocyanin production, resulting in the blushed skin of apricots [20]. *MdMYB1*, *MdMYB10*, and *MdMYBA* have the same effect on the fruit skin of apples (*Malus domestica*) [21,22,23]. Anthocyanin biosynthetic pathway has also been documented as closely associated with the transcription of *R2R3-MYBs* in tomatoes (*SlANT1*, *SlANT2*) [24], potatoes (*StAN1*, *StMYBA1*, *StMYB113*) [25,26], and eggplants (*SmMYB1*, *SmMYB75*) [27,28]. However, few studies focus on R2R3-MYBs related to anthocyanin biosynthesis for the leaf color formation of ornamental plants.

Recently, bioinformatic and molecular biology methods are more performed to identify R2R3-MYBs proteins from the genome in many species, which promotes a better understanding of gene families. In rice and Arabidopsis, 88 and 138 R2R3-MYBs were identified respectively [29]. Pineapple [30], lotus [31], and citrus [32] have also been studied. Additionally, natural evolution, duplication, and amplification of the genome contribute to the specificity of proteins in diverse organisms. A total of 36, 31, and 41 anthocyanin-related R2R3-MYBs have separately been characterized in *Actinidia chinensis* [33], *Gossypium hirsutum* [34], and *Brassica juncea* [35]. This kind of method is a popular and reliable tool to elucidate molecular regulations.

*A. comosus* var. *bracteatus*, belonging to the Bromeliaceae family, is a highly ornamental monocotyledon native to the tropical areas of South America and becomes an important fresh-cut flower around the world [36,37]. Since the accumulation of anthocyanin, the chimeric leaf, fruit, and bract of *A. comosus* var. *bracteatus* are red, while the flower is violet and the stem is purple (Figure S1). Therefore, *A. comosus* var. *bracteatus* is an excellent material for studying anthocyanin biosynthesis regulation mechanisms in different tissues and organs. We completed a high-quality genome sequencing and assembly of *A. comosus* var. *bracteatus* [38]. This provided a data basis for the genome-wide identification of the MYB gene family in *A. comosus* var. *bracteatus*. In the present work, all the R2R3-MYB transcription factors were identified in *A. comosus* var. *bracteatus*, and the *AbR2R3-MYB* genes with anthocyanin synthesis function were screened by bioinformatic analysis. The tissue-specific expression and hormone response characters of the anthocyanin biosynthesis-related *AbR2R3-MYB* genes were detected by RT-qPCR. The results will provide valuable insights into the role of AbR2R3-MYBs in the regulatory mechanisms of anthocyanin biosynthesis in *A. comosus* var. *bracteatus*.

## 2. Results

### 2.1. Identification and Characterization of AbR2R3-MYB Genes

To identify the R2R3-MYB proteins in *A. comosus* var. *bracteatus* genome, an HMM search was performed using the HMM profile of MYB binding domain, and R2R3-MYB proteins in *Arabidopsis* were used as a query in a local BLASTP against the genome. A total of 204 MYB sequences with MYB domains were identified. Pfam and NCBI CDD databases were employed to confirm the *R2R3-MYB* genes, and 99 typical *AbR2R3-MYB* genes were confirmed. Moreover, the basic information of 99 *AbR2R3-MYB* genes, including the length of coding sequences (CDS), amino acids, PIs, MWs, and subcellular localization, are listed in Table S1. The results revealed that the CDS length of the *AbR2R3-MYB* genes ranged from 396 bp to 2925 bp, and the lengths of the corresponding proteins were between 131 and 974 amino acids. The MWs ranged from 15.03 KDa to 108.60 KDa, and the theoretical pI values were between 4.79 and 10.44. The predicted GRAVY of *AbR2R3-MYB* proteins ranged from −1.054 to −0.265, which indicated that all the AbR2R3-MYB are hydrophilic proteins. All the AbR2R3-MYB were predicted to be localized in the nucleus, except for the Aco_HBLgroup9g006470, which was localized in the chloroplast.

### 2.2. Phylogenetic Analysis and Sequences Feature of AbR2R3-MYB Genes

**Figure 1:**
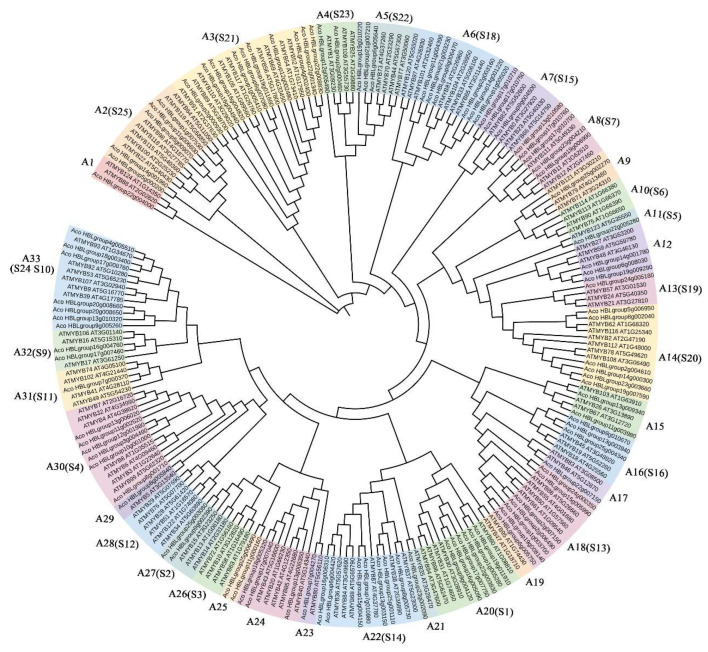
A neighbor-joining phylogenetic tree of R2R3-MYBs of *A*. *comosus* var. *bracteatus* and *Arabidopsis thaliana*.

In order to understand the evolution of AbR2R3-MYB, R2R3-MYB protein sequences of *A. comosus* var. *bracteatus* and *Arabidopsis* were used to construct a phylogenetic tree by ClustalW (Figure 1). The results indicated that these R2R3-MYB proteins could be grouped into 33 subfamilies based on the sequence similarity and topology, and S1 to S25 subfamilies in this tree were consistent with the previously well-characterized clades in *Arabidopsis* [1]. Noticeably, most subfamilies contained various members of R2R3-MYB from the two species. However, A10, A19, A26, and A28 clades were specific to *Arabidopsis*, while the A25 clade was specific to *A. comosus* var. *bracteatus.* To obtain insight into the conservation of the MYB domains, multiple sequence alignment of R2R3-MYBs from *A. comosus* var. *bracteatus* was performed (Figure S2). Generally, there are about 107 basic residues in the regions of R2R3-MYB domains from isolated R2R3-MYB proteins. Nevertheless, the length and amino acids compositions were highly varying in the outside regions of the domain. The R2 and R3 repeats contained evenly distributed and highly conserved Trp (W) residues. In the R2 repeat, three highly conserved Trp residues were located at positions 6, 27, and 47 (Figure S2A). In the R3 repeat, two Trp residues were located at positions 26 and 45, while the first Trp residues of most AbR2R3-MYBs were replaced by phenylalanine at position 4 (Figure S2B). We found a few insertions and deletions in five AbR2R3-MYBs (Figure S2C).

### 2.3. The Motif Composition and Gene Structure of AbR2R3-MYB Gene Family

**Figure 2:**
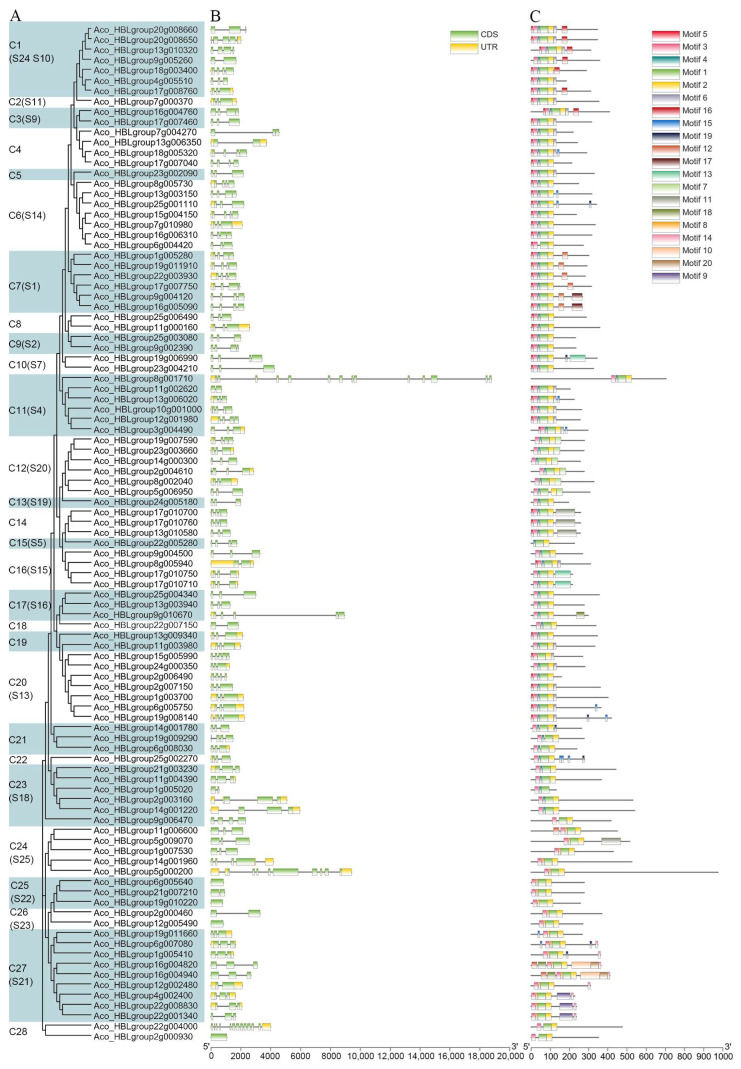
The motif composition and gene structure of *AbR2R3-MYB* gene family. (**A**) The neighbor-joining tree of 99 AbR2R3-MYB proteins. (**B**) Exon/intron structures of the *R2R3- MYB* genes. Yellow boxes, UTR; green boxes, exons; space between the boxes, introns. (**C**) The conserved motifs in the AbR2R3-MYB TFs were predicted using the MEME Suite web server.

The functions of gene members are strongly associated with their gene structure, which can reflect the phylogenetic relationships within gene families. Thus, the intron, exon boundaries, and conserved motifs of *AbR2R3-MYB* genes were investigated. The results showed that the number of introns in the coding sequences varied from one to five accounting for about 95% of *AbR2R3-MYB* genes (Figure 2B). However, there are four *AbR2R3-MYB* sequences were not disrupted by introns, and they have only one exon (*Aco_HBLgroup6g005640*, *Aco_HBLgroup19g010220*, *Aco_HBLgroup12g005490*, *Aco_HBLgroup2g000930*). In addition, a great proportion of *AbR2R3-MYB* genes (51%) contained a conserved gene structure with three exons and two introns. The *AbR2R3-MYB Aco_HBLgroup8g001710* included the highest number of introns (13) and exons (14). Although differential evolution among homologous genes might lead to various gene structures, the intron/exon structure of *AbR2R3-MYB* members from the same subgroups was highly conserved. This supported their close phylogenetic and evolutionary relationship. The phylogenetic tree was constructed based on AbR2R3-MYB protein sequences, which divided the AbR2R3-MYB gene family into 28 subgroups (Figure 2A). The topology structure was principally coherent with the above phylogenetic tree of *A. comosus* var. *bracteatus* and *Arabidopsis thaliana*.

Online program MEME was used to search for conserved motifs shared by these AbR2R3MYB proteins to further study the diversification of these *AbR2R3-MYB* genes. A total of 20 motifs were identified in the C-terminal regions and designated as motifs 1 to 20 (Figure 2C and Figure S3). Similar motifs were shared by the AbR2R3-MYB members within the same clade and high variance was observed among different subgroups, suggesting that the protein was conserved within a specific subfamily. We found that motif 16 was unique to the members of the C7 clade, and motif 11 was unique to the members of the C14 subgroup. These unique motifs may contribute to functional divergence. However, not all the members from the same subfamily contained the same motif types. For example, three genes in the C27 clade shared motif 9, but other members did not share it. Similarly, only one gene from the C24 clade contained motif 11, implying that it may have a different function from other gene members. These results indicated that sequence similarity could not necessarily group genes that are functionally like each other.

### 2.4. Chromosomal Distribution and Gene Duplication of AbR2R3-MYB Genes

**Figure 3:**
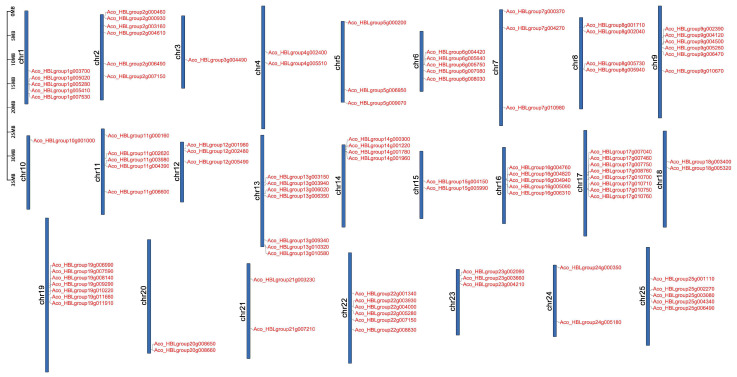
Chromosomal locations and distribution of *AbR2R3-MYB* genes. There are 25 chromosomes (Chr1–Chr25) in *A. comosus* var. *bracteatus*. Gene positions and chromosome length were measured using the scale on the left in megabases (Mb).

Genome chromosomal location analysis indicated that the *AbR2R3-MYB* genes were distributed across all 25 chromosomes among *A. comosus* var. *bracteatus* (Figure 3). All the 99 *AbR2R3-MYB* genes were mapped on linkage groups. Chromosome 17 contained the highest number of *AbR2R3-MYBs* with 8 *AbR2R3-MYBs*, followed by chromosomes 13 (7) and 19 (7), and six genes on chromosomes 2, 9, and 22. In contrast, only one gene was observed on chromosomes 3 and 10. High densities of genes were founded in the top and median parts of the chromosome. For instance, there were at least five genes in the median region of chromosomes 1, 6, 9, 16, 17, 19, and 22, and four genes in the top part of chromosomes 2 and 14.

**Figure 4:**
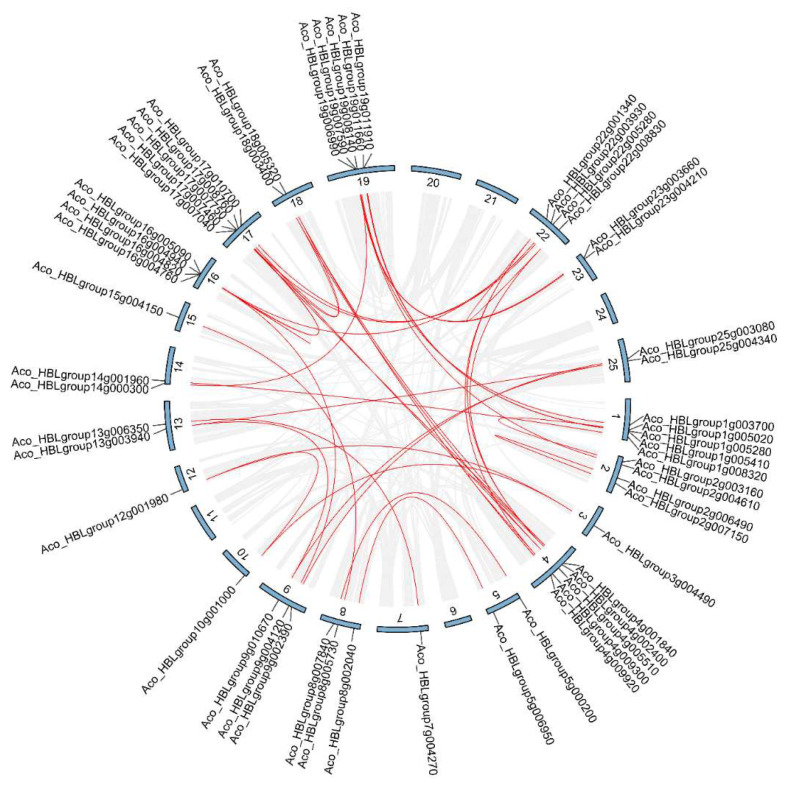
Gene duplication events of *AbR2R3-MYB* gene family in *A. comosus* var. *bracteatus*. Grey lines represent all synteny blocks in the genome, red lines suggest replicated *AbR2R3-MYB* gene pairs. The chromosome number is at the bottom of each chromosome.

Gene duplication, which generally occurs during plant evolution, plays an important role in the construction of new gene functions and has been recognized as a distinctive feature of plant genome evolution [39]. To study the potential duplication events of *MYB* genes in *A. comosus* var. *bracteatus*, the MCScanX (Multiple Collinearity Scan) method was used to determine the collinearity of the *AbR2R3-MYB* gene family. Finally, 32 segmental duplication events with 47 *R2R3-MYB* genes were identified in the *A*. *comosus* var. *bracteatus* genome (Figure 4). *AbR2R3-MYB* genes were located within synteny blocks on almost all chromosomes except numbers 6, 11, 20, 21, and 24. In this study, eight very closely related *AbR2R3-MYB* genes were physically located near each other in a syntenic region, forming four *AbR2R3-MYB* tandem duplication pairs (Table S2). The results showed that syntenic duplications mainly contribute to the expansion of *AbR2R3-MYB* genes. All the Ka, Ks, and Ka/Ks ratios of the above segmental and tandemly duplicated *AbR2R3-MYB* gene pairs are listed in Table S2. The Ka/Ks ratios of all the duplicated genes except for *Aco_HBLgroup16g004820* and *Aco_HBLgroup16g004940* were less than 1, implying that those had evolved under the pressure of purifying selection.

### 2.5. Analysis of the Cis-Acting Elements of the AbR2R3-MYB Genes

To better understand the functions of the *AbR2R3-MYB* genes in *A. comosus* var. *bracteatus*, we analyzed the cis-regulatory elements in the 2000 bp upstream promoter regions of *AbR2R3-MYB* genes using PlantCARE (Figure S4 and Table S3). A total of 30 light response elements, six stress tolerance elements, and 12 phytohormone responses elements were presented in the promoter regions. The light response element was dominant in all elements, especially in the Box element, with 409 distributed in 99 *AbR2R3-MYB* genes. Furthermore, 29 other kinds of light-responsive elements including 208 G-boxes, 138 GT1-motifs, 112 TCT-motifs, etc., were found in the promoter region, implying that *AbR2R3-MYBs* are widely involved in light-mediated regulation. In addition, many stress and phytohormone-responsive elements were detected. For example, LTR was involved in the response to low temperature, WUN-motif was related to wound response, and TC-rich repeats were involved in defense and stress responsiveness. P-box and GARE motifs were involved in response to gibberellin, CGTCA motifs and TGACG motifs were essential for MeJA responsiveness, and ABRE was involved in ABA responsiveness. Those abundant cis elements implicated that *AbR2R3-MYB* genes may be involved in the regulation of stress responses and hormone signaling pathways.

### 2.6. Protein Interaction Network

**Figure 5:**
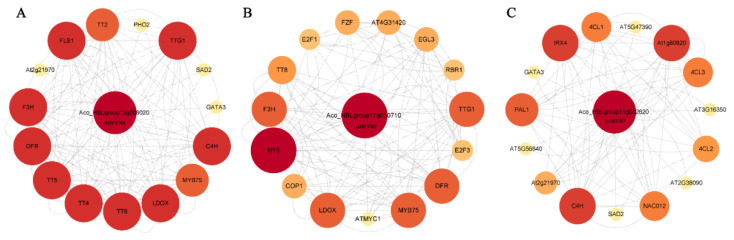
Protein interaction network of Aco_HBLgroup13g006020 (**A**), Aco_HBLgroup17g010710 (**B**), Aco_HBLgroup11g002620 (**C**) according to AbR2R3-MYB orthologs in *Arabidopsis*.

MYB family members generally perform the function by binding with target gene promoters or forming homo or heterodimers with other proteins [12]. We analyzed orthologous AtR2R3-MYB proteins and constructed a PPI network of the 99 AbR2R3-MYB protein candidates (Figure 5). This network was established from known interactions and predictions (based on neighboring genes, gene fusions, and gene co-occurrence) using various tools. We found that eighteen AbR2R3-MYB proteins did not interact with other proteins. Most of the AbR2R3-MYB bound with the promoter of genes involved in anthocyanin synthesis or interacted with MYC/TTG1 proteins in *Arabidopsis*. These proteins (especially Aco_HBLgroup13g006020, Aco_HBLgroup17g010710, and Aco_HBLgroup11g002620) were presumed to play important roles in regulating anthocyanin biosynthesis, whereas some AbR2R3-MYB proteins participated in plant growth and development.

### 2.7. Screening of the Anthocyanin-Related AbR2R3-MYB Genes by Phylogenetic Analysis

**Figure 6:**
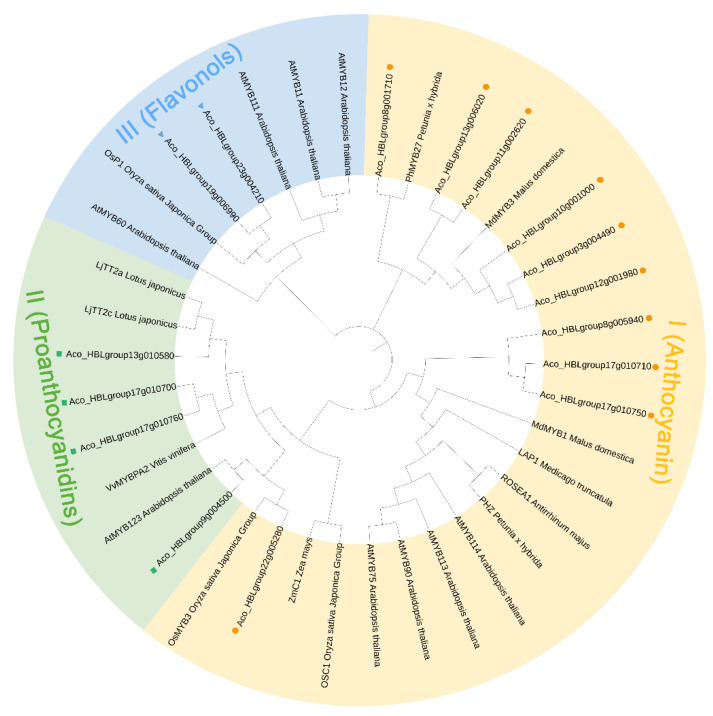
Phylogenetic analysis of the candidate AbR2R3-MYBs related to anthocyanin biosynthesis, flavonoids biosynthesis, and proanthocyanidin biosynthesis. The circles, squares, and triangles represent AbR2R3-MYBs in each branch.

To identify *AbR2R3-MYB* genes involved in the regulation of anthocyanin biosynthesis in *A. comosus* var. *bracteatus*, we constructed a phylogenetic tree that included all AbR2R3-MYBs along with 22 functional proteins related to flavonoid biosynthesis from other species (Figure S5). These AbR2R3-MYBs of anthocyanin, flavonoids, and proanthocyanidins clades were selected as candidates (Figure 6 and Figure S5). The proteins were divided into three subclades based on the proteins with known functions from other plants. Anthocyanin biosynthesis-related AbR2R3-MYB were concentrated in group I, containing 10 AbR2R3-MYB proteins (Aco_HBLgroup8g001710, Aco_HBLgroup22g005280, Aco_HBLgroup3g004490, Aco_HBLgroup17g010710, Aco_HBLgroup17g010750, Aco_HBLgroup10g001000, Aco_HBLgroup8g005940, Aco_HBLgroup13g006020, Aco_HBLgroup12g001980, and Aco_HBLgroup11g002620). They showed a high degree of homology with functional proteins involved in anthocyanin metabolism from *Arabidopsis thaliana*, *Petunia* × *hybrida*, *Antrirhinum majus*, *Medicago truncatula*, *Malus domestica*, *Zea mays*, and *Oryza sativa*. Therefore, these 10 *AbR2R3-MYB* genes potentially participated in anthocyanin synthesis. Similarly, there were two *AbR2R3-MYB* genes predicted to regulate flavonols and four genes might relate to proanthocyanidins biosynthesis.

### 2.8. Expression Patterns of AbR2R3-MYB Genes in Different Phenotypic Leaves

**Figure 7:**
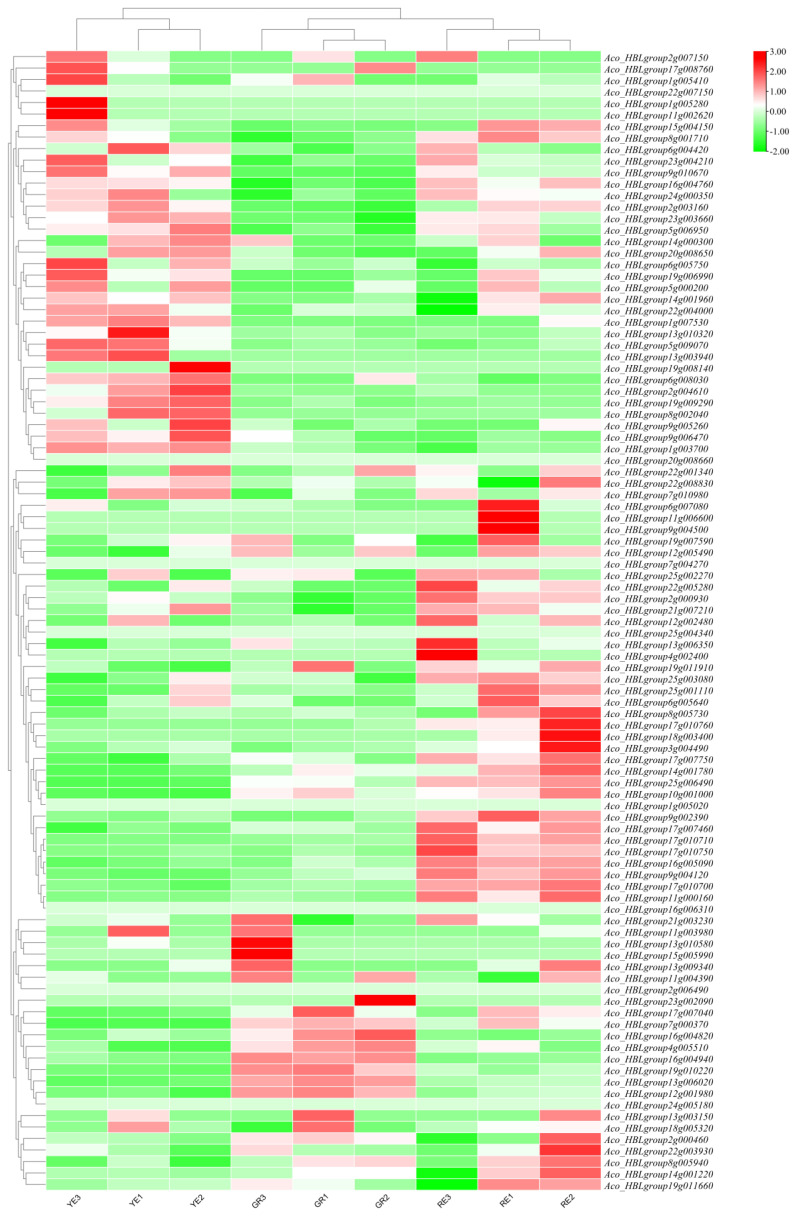
Expression patterns of *AbR2R3-MYB* genes in green leaves (GR), red leaves (RE), and yellow leaves (YE) of *A. comosus* var. *bracteatus*.

We compared the expression of *AbR2R3-MYB* genes in green leaves (GR), red leaves (RE), and yellow leaves (YE) of *A. comosus* var. *bracteatus* based on our published RNA-seq data (BioProject accession no. PRJNA720713) [40]. The transcripts of eight *AbR2R3-MYB* genes were not detected in these nine samples, which suggested that they were pseudogenes or poorly expressed in these samples (Figure 7). The *AbR2R3-MYB* genes were divided into five groups using hierarchical cluster analysis; these genes in the same subgroup could perform similar functions. We identified 38 *AbR2R3-MYB* genes including the candidate *AbR2R3-MYBs* associated with anthocyanin biosynthesis (*Aco_HBLgroup22g005280*, *Aco_HBLgroup3g004490*, *Aco_HBLgroup17g010710*, and *Aco_HBLgroup17g010750*) that showed higher expression in the red leaves compared to the green and yellow leaves, indicating that these genes may be responsible for the red phenotype of *A. comosus* var. *bracteatus* leaves.

### 2.9. Expressions of Anthocyanin Biosynthesis-Related Candidate AbR2R3-MYB Genes in Different Tissues

**Figure 8:**
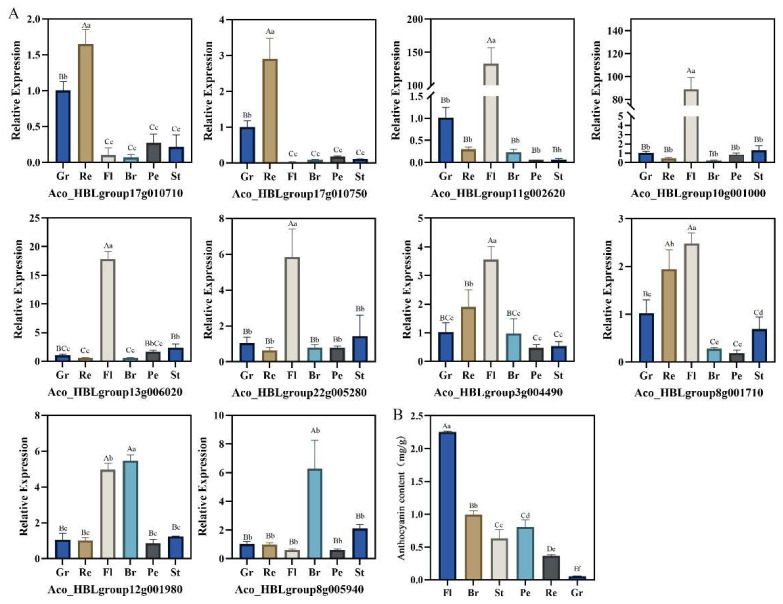
(**A**) The expression analysis of 10 anthocyanin biosynthesis-related candidate *AbR2R3-MYBs* in different tissues of *A. comosus* var. *bracteatus*. (**B**) Anthocyanin content in different tissues of *A. comosus* var. *bracteatus*. Gr, central green part of the chimeric leaf; Re, marginal red part of the chimeric leaf; Fl, flower; Br, bract; Pe, peel; St, stem bark. Different uppercase and lowercase letters indicate significant differences between different samples at the 0.01 and 0.05 levels, respectively.

The anthocyanin contents of different tissues were analyzed, and the results suggested that the highest anthocyanin content was detected in the flower (2.26 mg/g), and the bract was the second. The anthocyanin level of the leaf was the lowest (0.5 mg/g) (Figure 8B).

The expression levels of the 10 anthocyanin-related candidate *AbR2R3-MYB* genes in the flower, green leaf, red leaf, bract, peel, and stem bark were detected by RT-qPCR. (Figure 8A). The results showed that the expression of these genes had obvious tissue specificity. Two genes are expressed highest in the leaf, six genes in the flower, and two genes in the bract.

### 2.10. Expression of Anthocyanin Biosynthesis-Related Candidate AbR2R3-MYB to Hormone Treatments

**Figure 9:**
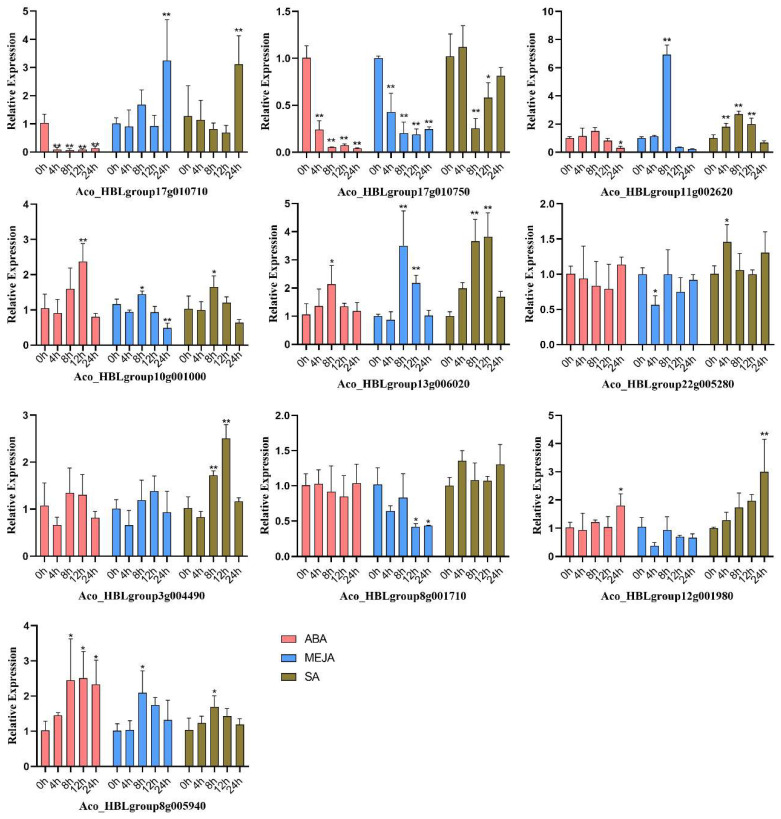
The relative expression levels of anthocyanin biosynthesis-related candidate *AbR2R3-MYB* genes under ABA, MEJA, and SA treatments. One asterisk and two asterisks indicate significant (*p* < 0.05) and extremely significant (*p* < 0.01) differences compared to the control, respectively.

Hormones are reported to regulate the expression of MYB transcriptional factors to affect the biosynthesis of anthocyanins, and are an important regulator to control the spatio-temporal accumulation of anthocyanin in plants [41]. ABA, SA, and MEJA response elements were widely identified in the promoter of these *AbR2R3-MYB* genes. RT-qPCR was conducted to reveal the effects of ABA, SA, and MEJA on the expression of these candidate *AbR2R3-MYB* genes. As shown in Figure 9, the results showed that six *AbMYB* genes were differentially expressed under at least one treatment, and most of them could be induced by multiple hormone treatments. Under ABA treatment, *Aco_HBLgroup10g001000 and Aco_HBLgroup8g005940* peaked at 12 h, *Aco_HBLgroup13g006020* peaked at 8 h, and *Aco_HBLgroup12g001980* peaked at 24 h. Under MEJA treatment, *Aco_HBLgroup11g002620, Aco_HBLgroup10g001000, Aco_HBLgroup13g006020,* and *Aco_HBLgroup8g005940* were induced more significantly at 8 h, exhibiting the higher expression difference compared to 0 h, and *Aco_HBLgroup17g010710* peaked at 24 h. Under SA treatment, *Aco_HBLgroup11g002620, Aco_HBLgroup10g001000,* and *Aco_HBLgroup8g005940* were induced remarkably at 8 h compared with 0 h. *Aco_HBLgroup17g010710* and *Aco_HBLgroup12g001980* expressed the highest at 24 h compared with 0 h. *Aco_HBLgroup13g006020* and *Aco_HBLgroup3g004490* peaked at 12 h, while *Aco_HBLgroup22g005280* peaked at 4 h.

## 3. Discussion

MYB transcription factors are important regulators involved in signaling and metabolic pathways. R2R3-MYB constitutes the largest MYB TF gene family in plants and plays an essential role in anthocyanin biosynthesis [42]. Previous studies have extensively identified the *R2R3-MYB* gene family in many species. Owing to the crucial roles of this anthocyanin-related gene family, the genome-wide study of R2R3-MYB has been conducted in model plants, as well as other species, such as sugar beet, citrus, lotus, cabbage, and pineapple. However, there was a lack of *R2R3-MYB* gene family study in *A. comosus* var. *bracteatus* as a popular ornamental plant. Recently, we have systematically reported the whole genome sequence of *A. comosus* var. *bracteatus* for the first time [38], providing support for screening the *R2R3-MYB* gene family. In the present study, we performed a genome-wide investigation of the *AbR2R3-MYB* gene family. A total of 99 *AbR2R3-MYB* genes were identified and divided into 33 subgroups (Figure 1). This result was a higher number of *AbR2R3-MYB* genes than that in pineapple (94) [30] but lower than those in lotus (*Nelumbo nucifera*) (116) [31], *Arabidopsis* (138) [29] and citrus (101) [32]. The different gene numbers of the same family in different species would be because of the size of the genome or evolution diversity [30,43].

The chromosome localization analysis of *AbR2R3-MYB* gene family members revealed that they were randomly distributed nonhomogeneously on the 25 chromosomes (Figure 3), which may be due to differences in chromosome structure and size. Gene duplication phenomenon widely exists in plants, and has an essential effect on genome evolution; we further performed the gene duplication analysis. If the gene pairs are located on different chromosomes, a gene duplication process could be considered segmental. In contrast, the duplication between genes on the same chromosome is called tandem duplication [44]. In our work, 32 gene pairs with 52 *R2R3-MYB* genes were identified (Table S2), most of which had been duplicated as a result of segment duplication, implying that the high segmental duplications played a crucial role in the expansion of the *AbR2R3-MYB* gene family (Figure 4). The gene copies produced by segmental duplication are often retained in the more slow-evolving MYB gene family [39], which was supported by a series of recent studies [4,29,45]. A large proportion of the segmental duplication events were identified in this study, which was also consistent with the evolutionary pattern of MYB genes. Duplication genes may undergo various selection processes: achieving nonfunctionalization through silencing, achieving neofunctionalization by getting new functions, or being subfunctionalized by dividing the original functions of ancestral genes [46,47,48]. In the present work, the expression levels of the identified tandem duplicated genes could be different in tissues. For instance, *Aco_HBLgroup2g006490* and *Aco_HBLgroup2g007150* were a pair of tandem duplicated genes, *Aco_HBLgroup2g006490* was not expressed, but *Aco_HBLgroup2g007150* highly expressed in yellow leaves (Figure 7). We speculated that the reason may be that *Aco_HBLgroup2g006490* lost the function of the original gene during evolution. The ratio between nonsynonymous (Ka) and synonymous (Ks) gene pairs is a good way to predict the selection method for the duplication process. It was found that the Ka/Ks values of all the *AbR2R3-MYB*s except one gene pair (*Aco_HBLgroup16g004820* and *Aco_HBLgroup16g004940*) were less than 1, suggesting that the majority of *AbR2R3-MYB* genes have undergone purification selection. It is worth noting that the Ka/Ks ratios greater than 1 show positive selection in duplication [49].

Structural characteristics of introns and exons can reveal phylogenetic relationships and are related to gene function [50]. An exon–introns structure analysis of the *AbR2R3-MYB* genes was performed and showed that clade C11 had more introns in this study (Figure 2A), implying that *AbR2R3-MYB* genes in clade C11 are relatively stable and evolutionarily conserved, which is conducive to the evolution of protein diversity [4,5]. It seems that the crucial function of the *R2R3MYB* gene family in *A. comosus* var. *bracteatus* is very much related to clade C11, and similar results were found in eggplant [28]. In addition to the similar gene structure, the motifs of the R2R3-MYB members in the same group were roughly the same. This result was in line with previous studies [30,32]. However, the gene structure and conserved motif in different groups were different, and we speculate that different groups may have different functions. These results confirm the characteristics of the *AbR2R3-MYB* gene family and facilitate further study on the function of *AbR2R3-MYB* genes.

*A. comosus* var. *bracteatus* is an important ornamental plant and is often used as a cut flower for decorating. Its various tissues and organs can appear red or purple, including chimeric leaves, bracts, flowers, peels, and stem bark, which is very rare in plants. Anthocyanin was reported to give plants colorations from red to purple [51]. The anthocyanin contents of different tissues of *A. comosus* var. *bracteatus* were detected, and it was found that the anthocyanin content in different tissues is in the following order: flower, bract, peel, stem bark, red part, and green part of chimeric leaves (Figure 8B). Thus, *A. comosus* var. *bracteatus* is an ideal material for the study of anthocyanins, and its regulatory mechanism deserves investigation. *R2R3-MYB* genes were widely reported to regulate anthocyanin biosynthesis [42]. To better understand the biological functions of *AbR2R3-MYB* genes, we analyzed their transcript abundance using transcriptome data of nine different leaf samples of *A. comosus* var. *bracteatus* and constructed a phylogenetic tree including the R2R3-MYB proteins from *A. comosus* var. *bracteatus* and several function-known R2R3-MYB proteins of other plant species. We selected genes from clade I (Figure 6) and detected their expression levels in different tissues. The transcripts of eight genes were not detected in all the leaf samples. A lack of expression data may indicate that they were pseudogenes or had special expression patterns not examined in our libraries. A hierarchical cluster analysis was performed using transcript data of other 91 *AbR2R3-MYB* genes (Figure 7). In most cases, genes presented in the same phylogenetic subgroup exhibited distinct expression patterns. However, closely related MYB genes (*Aco_HBLgroup17g010710* and *Aco_HBLgroup17g010750*) grouped together in the expression cluster, indicating that these genes could perform similar functions in red leaf formation. Some genes presented preferential expression across the different types of leaves. There was a total of 12 genes in yellow leaves, six genes in green leaves, and eight genes in red leaves. These genes could be involved in the regulation of biological processes in different types of leaves and were ideal candidates for functional analysis. Previous studies have shown that *MYBs* with close phylogenetic relationships have similar properties or functions [49]. For example, in *Lilium* spp., LhMYB6 and LhMYB12, a homolog of petunia AN2, determined organ and tissue-specific accumulation of anthocyanin by interacting with LhbHLH2 [15]. NnMYB5 from the *N. nucifera*, which is a homolog of EsAN2, induced the anthocyanin in flowers [17]. Therefore, based on phylogenetic analysis and RT-qPCR, we could obtain the functional information of *AbR2R3-MYB* genes by comparison with function-known MYB genes.

To investigate the function of the *AbR2R3-MYB* genes, a systematic analysis of the AbR2R3-MYB phylogeny of both *Arabidopsis* and *A. comosus* var. *bracteatus* was conducted. Most AbR2R3-MYBs clustered with orthologs from *Arabidopsis*. It is speculated that AbR2R3-MYBs in the same subgroup may possess common evolutionary origins and a conserved function. We found that Aco_HBLgroup23g004210 and Aco_HBLgroup19g006990 and AtMYB111/11/12 clustered together in the A8 subgroup (Figure 1), and previous studies have shown that overexpression of *AtMYB111* in tobacco-enhanced expression of genes of phenylpropanoid pathway leads to an elevated content of flavonols [52]. In addition, *AtMYB11* and *AtMYB12* were reported to enhance the biosynthesis of the flavonoids [53,54]. We also found that they were included in clade III (Figure 6), and all highly expressed in yellow leaves of *A. comosus* var. *bracteatus* (Figure 7). Therefore, we predicted that *Aco_HBLgroup23g004210* and *Aco_HBLgroup19g006990* may present similar biological functions in flavonols regulation, and are worthy of further functional study. In addition, we found that *Aco_HBLgroup17g010710* and *Aco_HBLgroup17g010750* were grouped into clade I (Figure 6), and had the same expression pattern, showing a high level of transcription in red leaves (Figure 7). The RT-qPCR result in Figure 8 confirmed the RNA-seq data, indicating that they have a similar function in regulating leaf anthocyanin. This kind of speculation is consistent with our previous study [40].

To investigate the function of the selected *AbR2R3-MYB* genes, the expression analysis by RT-qPCR was conducted in different tissues (Figure 8). We found that six genes expressed the highest in the flower, two genes in the bract, and two genes in the leaf, which was much more than in the pineapple [30]. *Aco_HBLgroup17g010710* and *Aco_HBLgroup17g10750* belonged to clade I, which are homologous genes of functional regulators of anthocyanin synthesis, such as *MdMYB1* from apple [22], *LAP1* from *Medicago* [55], and *AtMYB75*, *AtMYB90*, *AtMYB113*, and *AtMYB114* from *Arabidopsis* [18]. Moreover, RT-qPCR results showed that *Aco_HBLgroup17g010710* and *Aco_HBLgroup17g10750* were preferentially highly expressed in leaves. This was confirmed by the result of RNA-seq data and the PPI network (Figure 5). The results showed that *Aco_HBLgroup17g010710* and *Aco_HBLgroup17g10750* may share similar functions in the regulation of leaf anthocyanins, and we can verify this speculation through more in-depth experiments. *Aco_HBLgroup12g001980 and Aco_HBLgroup8g005940* were highly expressed in bracts, and have a close phylogenetic relationship with *MdMYB1* [22] and *MdMYB3* [56], indicating that these two *AbR2R3-MYB* genes could also be involved in the regulation of anthocyanin biosynthesis of bracts. In addition, *Aco_HBLgroup11g002620*, *Aco_HBLgroup10g001000, Aco_HBLgroup13g006020, Aco_HBLgroup22g005280, Aco_HBLgroup3g004490*, and *Aco_HBLgroup8g001710* showed high expression in the flowers and shared high sequence similarity with *PhMYB27*, *MdMYB3*, and *OsMYB3* [56,57,58], suggesting a similar functional feature in the anthocyanin regulation of flowers. In short, these genes may have similar functions that regulated the anthocyanin biosynthesis in corresponding tissues and can be candidate genes to improve ornamental quality; their specific functions will be determined in future studies.

Additionally, cis elements in the promoter can reveal the potential function of genes [59]. We analyzed cis elements in *AbR2R3-MYB* genes promoters and found that the identified cis elements in the promoter regions of these genes were mainly related to light, stress, and hormone responsiveness (Table S3). Some genes contained copies of cis elements, which may enhance the transcriptional regulation of genes and enable plants to adapt to changes in the environment. Interestingly, we found *AbR2R3-MYB* genes contain 30 types of light response, indicating that the transcriptions of most *AbR2R3-MYB* genes were induced by light. A total of 62 MBS elements under drought stress were detected in the promoter region, suggesting that these genes played an important role in response to pressures impressed by water limitation. A total of 273 ABRE responsiveness, 66 TCA-elements, 97 CGTCA-motifs, and TGACG-motifs were the main cis elements in the promoter region under response to ABA, SA, and MEJA. These results revealed the potential function of *AbR2R3-MYB* genes in response to hormone stress. To further identify candidate genes involved in the hormone signaling pathway, we measured their expression levels under hormones. The results showed that some selected *AbR2R3-MYB* genes responded to at least one treatment, and most of them could be induced by multiple treatments, suggesting that they may play a crucial role in the cross-talk among different signal transduction pathways in response to hormones. This is consistent with previous studies [30,60]. In addition, the results showed that the expressions of most *AbR2R3-MYB* genes were upregulated after hormone treatments, but their expression patterns were considerably different, implying that the signaling pathways in hormone response were complicated. *Aco_HBLgroup10g00100*, *Aco_HBLgroup12g001980,* and *Aco_HBLgroup8g005940* had the highest expression after ABA treatment. *Aco_HBLgroup17g010710, Aco_HBLgroup11g002620,* and *Aco_HBLgroup8g005940* were highly upregulated compared with 0 h under MEJA treatment. The expression levels of *Aco_HBLgroup13g006020, Aco_HBLgroup22g005280, Aco_HBLgroup3g004490,* and *Aco_HBLgroup12g001980* were high under SA treatment. These results were consistent with the prediction of its promoter cis-acting elements. However, not all genes that are induced to be highly expressed contained the corresponding cis elements in their promoters. For instance, *Aco_HBLgroup13g006020* did not contain ABA and MEJA responsive elements, but it was highly expressed after ABA and MEJA treatments for 8 h. *Aco_HBLgroup10g00100* lacked cis elements involved in MEJA and SA responsiveness, while it showed strong upregulation compared with 0 h after MEJA and SA treatment for 8 h. Similarly, *Aco_HBLgroup17g010710*, *Aco_HBLgroup11g002620*, and *Aco_HBLgroup8g005940* lacked SA responsive elements, whereas they were highly expressed after SA treatments for 24, 4, or 8 h. Thus, we speculate that the five genes responded by unknown regulative pathways to ABA, MEJA, or SA treatments. The same findings were found in *Camellia Sinensis* [61]. Moreover, ABA, MEJA, and SA are considered to be exogenous signals involved in the activation of anthocyanin synthesis in plants [62,63,64]. *MdbZIP44* interacts with *MdMYB1* to activate *MdMYB1*-mediated anthocyanin biosynthesis in response to ABA treatment. Likewise, these *AbR2R3-MYB* genes may exert functions in anthocyanin regulation responding to corresponding hormonal signals. In conclusion, we assume that certain *AbR2R3-MYB* genes could respond to different hormones and they might play important roles directly or indirectly in anthocyanin biosynthesis responding to hormones.

## 4. Materials and Methods

### 4.1. Identification of R2R3-MYB Genes in A. comosus var. bracteatus

The whole-genome protein sequences, annotation information, and genome sequences were obtained from NCBI. A hidden Markov model (HMM) of the Myb_DNA_binding domain (PF00249) downloaded from the Pfam database (http://pfam.xfam.org/ accessed on 18 October 2021) was performed for screening *MYB* genes by using an HMMER search (http://hmmer.org/download.html accessed on 18 October 2021) with a cutoff value of 0.01 and the default parameters. Moreover, R2R3-MYB protein sequences of *Arabidopsis* [29] were downloaded from TAIR (https://www.arabidopsis.org/ accessed on 18 October 2021) and were searched in BLASTP against the *A. comosus* var. *bracteatus* genome database (https://dataview.ncbi.nlm.nih.gov/object/PRJNA747096?reviewer=afs74oj8l0jcka2jibcsa2n3vf accessed on 18 October 2021). We merged the putative MYB proteins through the above methods, and further verified the presence of two repeats (R2 and R3) in all candidate sequences by the Pfam database and NCBI-CDD (https://www.ncbi.nlm.nih.gov/Structure/cdd/wrpsb.cgi accessed on 25 October 2021). At last, the amino acids, molecular weights (MWs), isoelectric points (PIs), and grand average hydropathicity (GRAVY) were predicted by the ExPASY website (https://web.expasy.org/protparam/ accessed on 25 October 2021). The WoLF PAORT program (https://wolfpsort.hgc.jp/ accessed on 25 October 2021) was used to examine the subcellular localization.

### 4.2. Phylogenetic Analysis and Sequence Analysis

Multiple sequence alignments of R2R3-MYB proteins from *A. comosus* var. *bracteatus* and *Arabidopsis* [29] were performed using the ClustalW program with default parameters and adjusted manually. MEGA 7.0 software was used to construct the unrooted neighbor-joining (NJ) phylogenetic tree [65]. The following paraments were employed: *P*-distance, pairwise deletion, and 1000 bootstrap replicates. To obtain a better understanding of the function of AbR2R3-MYB proteins, the full-length sequences of all the AbR2R3-MYB proteins, 16 selected AbR2R3-MYB proteins, and 22 R2R3-MYB proteins from other plants were used for phylogenetic analysis using the above methods. All the putative AbR2R3-MYB protein sequences were aligned by ClustalX using the default parameters. The GeneDoc software was used to display our results.

### 4.3. Gene Sructure and Conserved Motif Analysis of AbR2R3-MYB Genes

In order to understand the conservation of the *AbR2R3-MYB* genes, TBtools software was used to visualize the intron-exon distribution by analyzing the CDS and genome sequences [66]. Additionally, the conserved motifs of AbR2R3-MYB TFs were analyzed through the MEME Suite server (V 5.4.1; https://meme-suite.org/meme/tools/meme accessed on 27 December 2022), and the results were displayed by TBtools software. The optimum *E*-values were performed to identify the number of 20 motifs and the optimum width was set from 6 to 100 amino acids.

### 4.4. Chromosomal Distribution, Synteny Analysis, and the Selection Pressure of AbR2R3-MYB Genes

The chromosomal distribution of *AbR2R3-MYB* genes and their relative distances were analyzed using TBtools software. In order to analyze the duplication pattern of *AbMYB* genes, we used a genomic sequence annotation file [38] and the results were mapped by using TBtools. The TBtools toolkit software was applied to calculate the value of nonsynonymous (Ka) and synonymous (Ka) [66]. In addition, the MCScanX (Multiple Collinearity Scan) method was used to determine the collinearity of the *AbR2R3-MYB* gene family.

### 4.5. Analysis of the Cis Elements of AbR2R3-MYB

All the promoter sequences of candidate *AbR2R3-MYB* genes were submitted to the PlantCARE database (http://bioinformatics.psb.ugent.be/webtools/plantcare/html/ accessed on 27 December 2021) to predict the cis elements, and the results were adjusted manually. The TBtools software was used to graphically display the results.

### 4.6. Function Prediction of AbR2R3-MYBs

Online server STRING (V11.5, https://string-db.org/ accessed on 15 December 2022) was applied to construct the candidate AbR2R3-MYB protein association network. *A. thaliana* was set as the organism, and the genes with the highest bit scares were chosen to build the network. The interaction network was visualized by Cytoscape v3.9.1.

### 4.7. Expression Patterns of AbR2R3-MYB in the Different Pigmented Leaves

To explore the different patterns of the *AbR2R3-MYB* genes in different pigmented leaves of *A. comosus* var. *bracteatus*, the raw RNA-seq was obtained from the National Center for Biotechnology Information (NCBI) repository, and the BioProject accession number was PRJNA720713. We re-analyzed the raw data and constructed a comprehensive transcriptome. The expressions of *AbR2R3-MYBs* were normalized by calculating the transcripts per kilobase million (TPM) values. The expression profiles of the *AbR2R3-MYB* genes in various pigmented leaves were visualized by the TBtools software [66].

### 4.8. Plant Materials and Treatments

Five red representative tissues including flower, bract, peel, stem bark, and leaves were separately collected from the Experimental Station of Sichuan Agriculture University, Chengdu, China. Samples of three biological replicates were obtained from three comparison plants. The method of detecting the anthocyanin content was described in a previous study [67]. In order to investigate the expression patterns of the *AbR2R3-MYB* genes related to anthocyanin in response to hormone treatments, the following method was used. The green tissue culture plantlets were planted into plastic pots and kept in good condition. The plantlets with 10–12 expended leaves at the same development stages were adopted for hormone treatments. Samples were treated by ABA (abscisic acid, 100 μM), SA (salicylic acid, 100 μM), and MeJA (methyl jasmonate, 100 μM), respectively. Then, leaves were collected at 4, 8, 12, and 24 h after treatments. All the samples were stored at −80 °C for RNA extraction.

### 4.9. RNA Extraction and qRT-PCR Analysis

The LABGENE^TM^ Plant RNA Isolation Kit (Cat. No. LB1111, LABGENE Biotechnology Co., Ltd., Chengdu, China) was applied to isolate the total RNA of all the samples. RNA concentration and quality were measured by using NanoDrop 2000 spectrophotometer (Thermo Scientific, Waltham, MA, United States) and gel electrophoresis respectively. An amount of 1.0 μg good-quality RNA was used for cDNA synthesis by the *Evo M-MLV* RT Kit with gDNA Clean for qPCR II (Code No. AG11711, Accurate Biotechnology Co., Ltd., Changsha, China). Then, the cDNA was stored at −20 °C for further use. In this study, we used the premier 5.0 software to design premiers for real-time quantitative PCR (qRT-PCR), showing the sequences in detail in Table S4. qRT-PCR was performed on a CFX96 Real-time PCR Detection System with the SYBR^®^ Green Premix *Pro Taq* HS qPCR Kit (Cat No. AG11701, Accurate Biotechnology Co., Ltd., Changsha, China). The qRT-PCR program was set as follows: 95 °C for 30 s, and 40 cycles of 95 °C for 5 s, 58 °C for 20 s, and 72 °C for 30 s. Three biological replicates and technical replicates were performed in all experiments. The 2^−ΔΔCT^ method was used to analyze the relative expression levels [68]. *Unigene.16454* and *Unigene.16459* were used as the internal reference genes for different tissues, and *IDH* and *SDP* were used as reference genes for different hormone treatments [69].

## 5. Conclusions

Based on the whole genome information of *A. comosus* var. *bracteatus*, we performed a comprehensive analysis of the AbR2R3-MYB gene family. A total of 99 full-length *R2R3-MYB* genes were identified and phylogenetically divided into 33 subfamilies, which were supported by the conserved gene structures and motifs. These *AbR2R3-MYB* genes are unevenly distributed among 25 chromosomes in *A. comosus* var. *bracteatus.* Synteny analysis suggested that the segmental duplication events led to the expansion of the AbR2R3-MYB gene family. Most gene pairs of AbR2R3-MYB proteins had evolved under the strong pressure of purifying selection. In addition, the anthocyanin regulator candidates were identified based on the phylogenomic results. The expression analysis contributed to the assignment of tissue preference and hormone expression patterns of the candidate genes. These results revealed the possible functions of *AbR2R3-MYB* genes in regulating anthocyanin of different tissues and response to hormones. Our study initiated a better understanding of AbR2R3-MYB gene family characteristics and provided valuable information that facilitates further analysis of anthocyanin regulation and hormone responses of *A. comosus* var. *bracteatus*.

## Data Availability

Not applicable.

## Supplementary Materials

The following supporting information can be downloaded at: https://www.mdpi.com/article/10.3390/ijms24043133/s1.
