# Supplementary material for: Genome-Wide Identification and Characterization of R2R3-MYB Provide Insight into Anthocyanin Biosynthesis Regulation Mechanism of Ananas comosus var. bracteatus
Source: Int J Mol Sci. 2023 Feb 5;24(4):3133. doi: 10.3390/ijms24043133 (PMC9964748; doi:10.3390/ijms24043133)
Supplement: Supplementary file 1 [file ijms-24-03133-s001.zip › Supplementary Files in one PDF.pdf]

Supplementary Material

Supplementary Figures

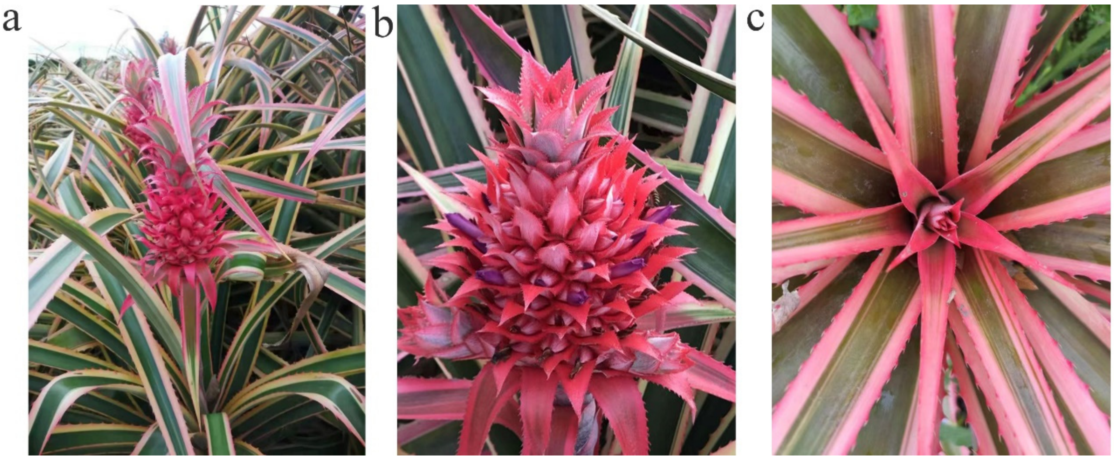

Supplementary Figure S1. The red Phenotype of different tissues in *Ananas comosus* var. *bracteatus*

A

```
                                              Helix 1        Helix 2        Helix 3
                                         *         20         *         40         *
Aco_HBLgroup6g007080/76-348   : CARGHWRPAEDAKLKELVSQYG-PQNWNLIAEKLQGRS-GKSCRLRWFNQLDPRINR :  55
Aco_HBLgroup14g001960/35-500  : LVKGSWTHEEDNKLIHLVKKHG-QSKWSIIAN-FLPGRIGKQCRERWHNHLNPEIKK :  55
Aco_HBLgroup19g007590/21-278  : LRRGPWTVEEDLILVNYITAHG-EGRWNSLARCAGLKRTGKSCRLRWLNYLRPDVRR :  56
Aco_HBLgroup13g006020/14-225  : -NKGAWTKEEDERLIAHIREHG-EGCWRALPKAAGLLRCGKSCRLRWINYLRPDLKR :  55
Aco_HBLgroup19g006990/12-344  : LKKGRWTEEEDEILVKYITTHG-EGSWRSLPKNAGLLRCGKSCRLRWINYLRADLKR :  56
Aco_HBLgroup8g005940/34-310   : VKRGPWTADEDEVLSSYVRREG-EGRWRTLPKRAGLLRCGKSCRLRWMNYLRPSIKR :  56
Aco_HBLgroup11g000160/14-358  : LNKGSWTPEEDMRLIAYIHKYG-HGNWRALPKQAGLLRCGKSCRLRWINYLRPDIKR :  56
Aco_HBLgroup13g006350/12-241  : VKRGPWTAEEDKKLIGFLL-THGHCCWRAVPKLAGLLRCGKSCRLRWTNYLRPDLKR :  56
Aco_HBLgroup2g006490/12-158   : LRKGLWSPEEDEKLLKHITKYG-HGCWSSVPKLAGLQRCGKSCRLRWINYLRPDLKR :  56
Aco_HBLgroup22g003930/16-284  : --KGPWTPEEDITLVSYIQEHG-PGNWRAVPTQTGLMRCSKSCRLRWTNYLRPGIRR :  54
Aco_HBLgroup17g010760/12-258  : QNKGSWSVSEDKILTEYIKLHG-EGKWRDLPTRAGLKRCGKSCRLRWMNYLRPGIKR :  56
Aco_HBLgroup1g007530/122-428  : VVKGQWTAEEDRLLVQLVDQHG-VRRWSQIAKNLVGRI-GKQCRERWHNHLRPDIKK :  55
Aco_HBLgroup15g004150/12-235  : VKRGPWSPEEDAILRSYVEMYGTGGNWIALPQKAGLKRCGKSCRLRWLNYLRPNIKH :  57
Aco_HBLgroup16g006310/12-316  : VKKGPWSPEEDAKLKAYIEEHGTGGNWIALPQKIGLKRCGKSCRLRWLNYLRPNIKH :  57
Aco_HBLgroup5g000200/70-513   : LVKGPWSKQEDEIIIQMVKKYG-PKKWSTISQALPGRI-GKQCRERWHNHLNPAINK :  55
Aco_HBLgroup13g010320/60-313  : --KGPWTPEEDKLLVEYIQKNG-HGSWRHLPKLAGLNRCGKSCRLRWTNYLRPDIKR :  54
Aco_HBLgroup11g004390/23-325  : LKKGPWTAAEDEILTEYVRRHG-EGNWNAVRRNTGLLRCGKSCRLRWMNHLRPDLKK :  56
Aco_HBLgroup4g005510/16-185   : --KGPWTPEEDQKLIQYIQKHG-HGSWRALPKLAGLNRCGKSCRLRWTNYLRPDIKR :  54
Aco_HBLgroup4g002400/2-228    : CTRGHWRPSEDEKLKELVACYG-PHNWNAIAEKLQGRS-GKSCRLRWFNQLDPRINR :  55
Aco_HBLgroup22g007150/28-338  : LRKGLWSPEEDDKLMSYMLSNG-QGCWSDVARNAGLQRCGKSCRLRWINYLRPDLKR :  56
Aco_HBLgroup12g002480/16-310  : CPRGHWRPGEDEKLRQLVEQYG-PQNWNSIAEKLQGRS-GKSCRLRWFNQLDPRINK :  55
Aco_HBLgroup19g011910/16-294  : --KGPWTPEEDIILVSYIQEHG-PGNWRSVPTNTGLMRCSKSCRLRWTNYLRPGIKR :  54
Aco_HBLgroup15g005990/12-269  : LRKGLWSPEEDEKLYNHIIRH-------------GLQRCGKSCRLRWINYLRPDLKR :  44
Aco_HBLgroup24g005180/13-195  : VRKGPWTLEEDLILINYITHHG-EGVWNNLARSAGLNRTGKSCRLRWLNYLRPDVRR :  56
Aco_HBLgroup12g001980/14-256  : -NKGAWTKEEDQRLIAYIQAHG-EGCWRSLPKAAGLLRCGKSCRLRWINYLRPDLKR :  55
Aco_HBLgroup17g007750/16-316  : --KGPWTPEEDLMLVSYIQEHG-PGNWRSVPANTGLMRCSKSCRLRWTNYLRPGIKR :  54
Aco_HBLgroup13g003940/13-276  : YRKGLWSPEEDQRLRDYILSHG-LSCWSAVPAKAGLQRNGKSCRLRWINYLRPGLKR :  56
Aco_HBLgroup11g006600/155-450 : VVKGQWTLEEDRLLINLVEQYG-VRRWSQIAQMLPGRI-GKQCRERWHNHLRPNIKK :  55
Aco_HBLgroup18g003400/16-290  : --KGPWTPEEDQKLVQHIQAHG-HGSWRALPKLAGLNRCGKSCRLRWTNYLRPDIKR :  54
Aco_HBLgroup13g009340/12-345  : VKRGLWSPEEDEKLIRYITTHG-YGCWSEVPEKAGLQRCGKSCRLRWINYLRPDIRR :  56
Aco_HBLgroup16g004820/84-364  : CVRGHWRPAEDSKLRELVALYG-PQNWNLIAEKLEGRS-GKSCRLRWFNQLDPRINR :  55
Aco_HBLgroup23g002090/12-329  : VKKGPWTAEEDAKLLAYTSTHGTG-NWTSVPQKAGLKRCGKSCRLRYTNYLRPNLKH :  56
Aco_HBLgroup6g005640/3-277    : RIKGPWSPEEDELLQRLVQEHG-ARNWSLISRSIPGRS-GKSCRLRWCNQLSPQVEH :  55
Aco_HBLgroup10g001000/14-264  : -NKGAWTKEEDQRLIAYIKAHG-EGCWRSLPKAAGLLRCGKSCRLRWMNYLRPDLKR :  55
Aco_HBLgroup1g005280/16-303   : --KGPWTPEEDIILVSYIQEHG-PGNWRSVPTNTGLMRCSKSCRLRWTNYLRPGIKR :  54
Aco_HBLgroup23g003660/14-275  : LRRGPWSVEEDILLMNYIAIHG-EGRWNSLARCAGLKRTGKSCRLRWLNYLRPDVRR :  56
Aco_HBLgroup11g003980/11-332  : VKRGLWSPEEDEKLIKHITTHG-HNCWSSVPKLAGLQRCGKSCRLRWINYLRPDLKR :  56
Aco_HBLgroup5g009070/171-514  : VVKGQWTLEEDRRLIKLVEQHG-LRKWSHIAQMLPARI-GKQCRERWHNHLRPNIKK :  55
Aco_HBLgroup19g011660/64-266  : CSRGHWRPAEDARLRELVSQFG-PQNWNLIAEKLDGRS-GKSCRLRWFNQLDPRINK :  55
Aco_HBLgroup3g004490/53-297   : -NKGAWTKEEDERLVAYIKAHG-EGCWRSLPKAAGLLRCGKSCRLRWINYLRPDLKR :  55
Aco_HBLgroup13g010580/12-256  : LNRGAWTSHEDKLLSEYIMAHG-LGRWRSLPADAGLNRCGKSCRLRWLNYLRPDIKR :  56
Aco_HBLgroup7g004270/12-219   : VKRGPWTAQEDKRLAEFIAADGRRCCWRAVPKLAGLQRCGKSCRLRWTNYLRPGLNH :  57
Aco_HBLgroup7g000370/16-355   : --RGPWTPEEDQKLVEYIRKNG-QGNWRTLPKNAGLARCGKSCRLRWTNYLRPDIKR :  54
Aco_HBLgroup22g005280/1-225   : ------------MLVSYITAHG-EGKWGSLPKRAGLKRCGKSCRLRWLNYLRPGIKR :  44
Aco_HBLgroup8g001710/421-705  : -DKGVSSKPEDEKLAECIRVHG-EGCWRSLPRAAGLLWCGKSCRIRWINYLRPDEKR :  55
Aco_HBLgroup17g010700/12-258  : QNKGAWSVSEDKILTEYIKLHG-EGKWRDLPTRAGLKRCGKSCRLRWMNYLRPGIKR :  56
Aco_HBLgroup25g003080/12-231  : LKKGPWTPEEDQILISYIQKYG-HENWRALPKLAGLLRCGKSCRLRWINYLRPDIKR :  56
Aco_HBLgroup18g005320/12-290  : LKKGPWTAEEDKKLINFIL-NNGQCCWRAVPKLAGLLRCGKSCRLRWTNYLRPDLKR :  56
Aco_HBLgroup1g005410/64-362   : CCRGHWRPVEDAKLRELVSQFG-PQNWNLIAENLQGRS-GKSCRLRWFNQLDPRINK :  55
Aco_HBLgroup17g008760/16-313  : --KGPWTPEEDQKLVDFIQKHG-HGSWRALPKLAGLNRCGKSCRLRWTNYLRPDIKR :  54
Aco_HBLgroup17g007040/12-212  : LKKGPWTEEEDKKLVEFILSNNGHCCWRAVPKLAGLLRCGKSCRLRWTNYLRPDLKR :  57
Aco_HBLgroup9g002390/12-233   : VKKGPWTPEEDRILVDYIQRYG-HGNWRALPKQAGLLRCGKSCRLRWTNYLRPDIKR :  56
Aco_HBLgroup11g002620/14-204  : -NKGAWTKEEDDRLVAYIREHG-EGCWRSLPKAAGLLRCGKSCRLRWINYLRPDLKR :  55
Aco_HBLgroup7g010980/12-334   : VKKGPWSPEEDAKLKSYIEQHGTGGNWIALPQKIGLKRCGKSCRLRWLNYLRPNIKH :  57
Aco_HBLgroup8g002040/20-328   : LRRGPWTLEEDELLMRYIAYHG-EGRWNLLARCSGLKRTGKSCRLRWLNYLKPDIKR :  56
Aco_HBLgroup13g003150/12-316  : VKKGPWSPEEDRKLKEYIETYGTGGNWIALPQKAGLKRCGKSCRLRWLNYLRPNIKH :  57
Aco_HBLgroup8g005730/12-248   : VKRGPWSPEEDDALRNYVEKHGSGGSWISLPQKAGLKRCGKSCRLRWLNYLRPDIKH :  57
Aco_HBLgroup9g005260/17-360   : --KGPWTAEEDKKLVEYIEKHG-HGNWRSLPTSAGLNRCGKSCRLRWTNYLRPDIKR :  54
Aco_HBLgroup1g003700/12-401   : LRKGLWSPEEDEKLLKHITKYG-HGCWSSVPKLAGLQRCGKSCRLRWINYLRPDLKR :  56
Aco_HBLgroup16g004940/133-411 : CVRGHWRPAEDSKLRELVALYG-PQNWNLIAEKLEGRS-GKSCRLRWFNQLDPRINR :  55
Aco_HBLgroup17g010750/12-215  : LSRGKWTEEEDRILASYVEAHG-VENWSKLPMKAGLNRSGKSCRLRWLNYLRPNIKR :  56
Aco_HBLgroup19g008140/12-418  : LRKGLWSPEEDEKLINHITKYG-HGCWSSVPKLAGLQRCGKSCRLRWINYLRPDLKR :  56
Aco_HBLgroup22g001340/2-235   : CTRGHWRPSEDEKLKELVARYG-PHNWNAIAEKLQGRS-GKSCRLRWFNQLDPRINR :  55
Aco_HBLgroup25g001110/12-341  : VKKGPWSPEEDAKLKEFIDKYGTGGNWIALPQKAGLKRCGKSCRLRWLNYLRPNLKH :  57
Aco_HBLgroup25g004340/13-355  : CRKGLWSPEEDRKLRDYILRYG-HGCWSVVPAKAGLQRNGKSCRLRWINYLRPGVKR :  56
Aco_HBLgroup16g004760/79-411  : --KGPWTPEEDQKLLAYIEEHG-HGCWRALPAKAGLQRCGKSCRLRWTNYLRPDIKR :  54
Aco_HBLgroup25g006490/14-288  : LNKGSWTQDEDARLIAYIQKHG-HGNWRALPKKAGLLRCGKSCRLRWINYLRPDIKR :  56
Aco_HBLgroup22g004000/31-467  : RHIVTWTPQEDDLLREQIALHG-TDNWTSIAAQFKDKT-SRQCRRRWYTYLNSECKK :  55
Aco_HBLgroup14g001220/40-460  : LKKGPWTSAEDAILIDYVKKHG-EGNWNAVQKNSGLSRCGKSCRLRWANHLRPNLKR :  56
Aco_HBLgroup21g007210/3-276   : RIKGPWSPEEDEALQRLVQKHG-ARNWSLISKSIPGRS-GKSCRLRWCNQLSPQVEH :  55
Aco_HBLgroup12g005490/42-270  : GSKVPWSPHEDKMLRALVKKHG-AQSWSIICRNIPGRS-AKSCRLRWCNHLLPGLNF :  55
Aco_HBLgroup2g003160/39-479   : LKKGPWTADEDSILSEYVEKFG-EGNWNNVQKTTGLARNGKSCRLRWANHLRPNLKK :  56
Aco_HBLgroup9g010670/13-298   : YRKGLWSPEEDQRLRDYILRYG-HGCWSSVPAKAGLQRNGKSCRLRWINYLRPGLKR :  56
Aco_HBLgroup14g000300/3-257   : LRRGPWTMEEDLILINYIANHG-EGRWNSLARSAGLKRTGKSCRLRWLNYLRPDVRR :  56
Aco_HBLgroup20g008650/16-347  : --KGPWTPEEDKLLTDYIQQNG-HGSWRRLPKLAGLNRCGKSCRLRWTNYLRPDIKR :  54
Aco_HBLgroup22g008830/2-235   : CTRGHWRPSEDEKLKELVARYG-PHNWNAIAEKLQGRS-GKSCRLRWFNQLDPRINR :  55
Aco_HBLgroup19g010220/10-257  : RIKGSWSPEEDAMLTQLVKDHG-ARNWSLISDGIPGRS-GKSCRLRWCNQLSPDVHH :  55
Aco_HBLgroup2g000460/60-369   : RVKGPWSPEEDAVLSRLVSKFG-ARNWSLIARGIPGRS-GKSCRLRWCNQLDPYVKR :  55
Aco_HBLgroup9g004500/23-269   : VNRGAWTAEEDQKLVEYVRLHG-DKKWRTLPAKAGLNRCGKSCRLRWLNYLRPGIKR :  56
Aco_HBLgroup21g003230/25-397  : MKKGPWTAAEDAILVDYVRKHG-EGNWNAVQRNSGLARCGKSCRLRWANHLRPNLKK :  56
Aco_HBLgroup9g004120/16-270   : --KGPWTPEEDLMLVSYIQEHG-PGNWRAVPTNTGLMRCSKSCRLRWTNYLRPGIRR :  54
Aco_HBLgroup25g002270/16-279  : WRKGPWTSEEDKLLMQHVNQHG-EGRWNSVSKLTGLRRSGKSCRLRWVNYLRPDLKK :  56
Aco_HBLgroup6g005750/12-364   : LRKGLWSPEEDEKLINHIAKYG-HGCWSSVPKQAGLQRCGKSCRLRWINYLRPDLKR :  56
Aco_HBLgroup6g004420/12-272   : VKKGPWSPEEDAKLKVYIEEHGTGGNWIAPPYKIGIKRCGKSCRLRWLNYLRPNIKH :  57
Aco_HBLgroup2g007150/12-361   : LRKGLWSPEEDEKLLKHITKYG-HGCWSSVPKLAGLQRCGKSCRLRWINYLRPDLKR :  56
Aco_HBLgroup23g004210/12-325  : LKKGRWSKEEDEILVKYISANG-EGSWRSLPKNAGLLRCGKSCRLRWINYLRADLKR :  56
Aco_HBLgroup16g005090/16-270  : --KGPWTPEEDLMLVSYIQEHG-PGNWRAVPTNTGLMRCSKSCRLRWTNYLRPGIRR :  54
Aco_HBLgroup9g006470/111-416  : LRKGPWMAEEDEILLEYVRSHG-PRDWSSIRSKGLLPRTGKSCRLRWVNKLKPDLKT :  56
Aco_HBLgroup2g004610/45-277   : LRRGPWTVDEDLMLINYIANHG-GGQWNSLARSAGLKRTGKSCRLRWLNYLRPDVRR :  56
Aco_HBLgroup17g010710/12-215  : LSRGKWTEEEDRILASYVEAHG-AENWAKLPIKAGLNRCGKSCRLRWLNYLRPNIKR :  56
Aco_HBLgroup20g008660/17-347  : --KGPWTPEEDRLLKECVEKHG-NGSWRRVPKLAGLNRCGKSCRLRWTNYLRPGIKR :  54
Aco_HBLgroup24g000350/13-281  : LRKGLWSPEEDEKLYNHISRFG-VGSWSSVANLAGLQRCGKSCRLRWINYLRPDLKR :  56
Aco_HBLgroup17g007460/16-317  : --KGPWTPEEDQKLLAYIEEHG-HGSWRALPAKAGLQRCGKSCRLRWTNYLRPDIKR :  54
Aco_HBLgroup1g005020/19-131   : LRKGPWTAEEDRILREYVTKHG-EGNWNVVQKNSGLSRCGKSCRLRWINHLRPDLKK :  56
```

B

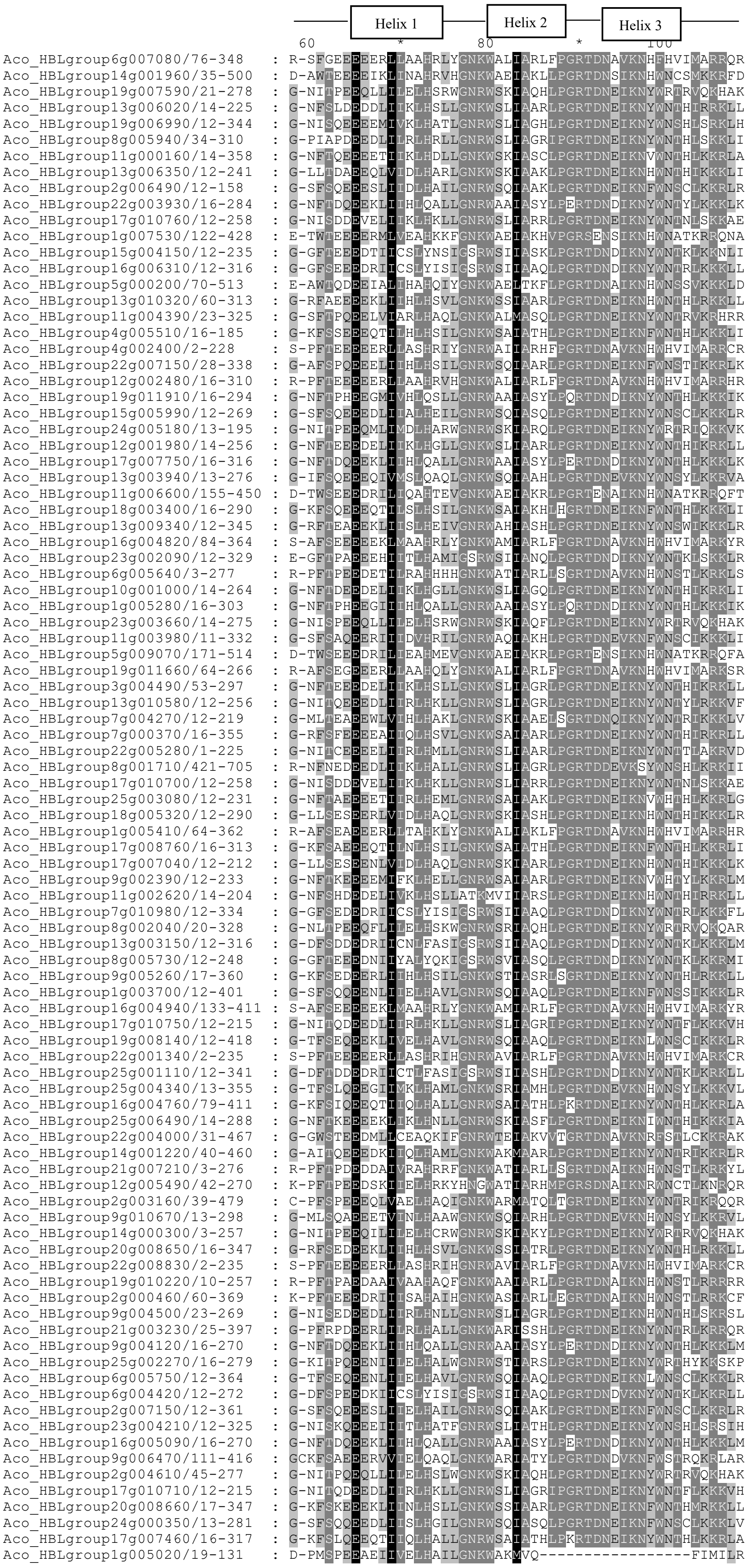
Helix 1
Helix 2
Helix 3
60 * 80 * 100
Aco_HBLgroup6g007080/76-348 : R-SFGEEEEERLLAAHRLYGNKWALIARLFPGRTDNAVKNHFHVIMARRQR
Aco_HBLgroup14g001960/35-500 : D-AWTEEEEIKLLNAHRVHGNKWAEIAKLLPGRTDNSIKNHWNCSMKKRFD
Aco_HBLgroup19g007590/21-278 : G-NITPEEQLLILELHSRWGNRWSKIAQHLPGRTDNEIKNYWRTRVQKHAK
Aco_HBLgroup13g006020/14-225 : G-NFSLDEEDDLIIKLHSLLGNKWSLIAARLPGRTDNEIKNYWNTHIRRKLL
Aco_HBLgroup19g006990/12-344 : G-NISQEEEEMIVKLHATLGNKWSLIAGHLPGRTDNEIKNYWNSHLSRKLH
Aco_HBLgroup8g005940/34-310 : G-PIAPDEEDLIIRLHRLLGNRWSLIAGRIPGRTDNEIKNYWNTHLSKKLI
Aco_HBLgroup11g000160/14-358 : G-NFTQEEEETIIKLHDLLGNKWSKIASCLPGRTDNEIKNVWNTHLKKRLA
Aco_HBLgroup13g006350/12-241 : G-LLTDAEEQLVIDLHARLGNKWSKIAAKLPGRTDNEIKNHWNTHIKKKLI
Aco_HBLgroup2g006490/12-158 : G-SFSQEEESLIIDLHAILGNRWSQIAAKLPGRTDNEIKNFWNSCLKKRLR
Aco_HBLgroup22g003930/16-284 : G-NFTDQEEKLIIHLQALLGNRWAAIASYLPERTDNDIKNYWNTYLKKKLK
Aco_HBLgroup17g010760/12-258 : G-NISDDEVELIIKLHKLLGNRWSLIARRLPGRTDNEIKNYWNTNLSKKAE
Aco_HBLgroup1g007530/122-428 : E-TWTEEEERMLVEAHKKFGNKWAEIAKHVPGRSENSIKNHWNATKRRQNA
Aco_HBLgroup15g004150/12-235 : G-GFTEEEDTIICSLYNSIGSRWSIIASKLPGRTDNDIKNYWNTKLKKNLI
Aco_HBLgroup16g006310/12-316 : G-GFSEEEDRIICSLYISIGSRWSIIAAQLPGRTDNDIKNYWNTRLKKKLL
Aco_HBLgroup5g000200/70-513 : E-AWTQDEEIALIHAHQIYGNKWAELTKFLPGRTDNAIKNHWNSSVKKKLD
Aco_HBLgroup13g010320/60-313 : G-RFAEEEEKLIIHLHSVLGNKWSSIAARLPGRTDNEIKNHWNTHLRKKLL
Aco_HBLgroup11g004390/23-325 : G-SFTPQEEELVIARLHAQLGNKWALMASQLPGRTDNEIKNYWNTRVKRHRR
Aco_HBLgroup4g005510/16-185 : G-KFSSEEEQTIIHLHSILGNKWSAIATHLPGRTDNEIKNFWNTHLKKKLI
Aco_HBLgroup4g002400/2-228 : S-PFTEEEEERLLASHRIYGNRWAIIARHFPGRTDNAVKNHWHVIMARRCR
Aco_HBLgroup22g007150/28-338 : G-AFSPQEEELIIHLHSILGNRWSQIAARLPGRTDNEIKNFWNSTIKKRLK
Aco_HBLgroup12g002480/16-310 : R-PFTEEEERLLAAHRVHGNKWALIARLFPGRTDNAVKNHWHVIMARRHR
Aco_HBLgroup19g011910/16-294 : G-NFTPHEEGMIVHLQSLLGNRWAAIASYLPQRTDNDIKNYWNTHLKKKIK
Aco_HBLgroup15g005990/12-269 : G-SFSQEEEDLIIALHEILGNRWSQIASQLPGRTDNEIKNYWNSCLKKKLR
Aco_HBLgroup24g005180/13-195 : G-NITPEEQMLIMDLHARWGNRWSKIARQLPGRTDNEIKNYWRTRIQKKVK
Aco_HBLgroup12g001980/14-256 : G-NFTEEEDELIIKLHGLLGNKWSLIAARLPGRTDNEIKNYWNTHIKRKLL
Aco_HBLgroup17g007750/16-316 : G-NFTDQEEKLIIHLQALLGNRWAAIASYLPERTDNDIKNYWNTHLKKKLK
Aco_HBLgroup13g003940/13-276 : G-IFSQEEEQIVMSLQAQLGNKWSQIAAHLPGRTDNEVKNYWNSYLKKRVA
Aco_HBLgroup11g006600/155-450 : D-TWSEEEDRILIQAHTEVGNKWAEIAKRLPGRTENAIKNHWNATKRRQFT
Aco_HBLgroup18g003400/16-290 : G-KFSQEEEQTIISLHSILGNKWSAIAKHLHGRTDNEIKNFWNTHLKKKLI
Aco_HBLgroup13g009340/12-345 : G-RFTEAEEKLIISLHEIVGNRWAHIASHLPGRTDNEIKNYWNSWIKKKLR
Aco_HBLgroup16g004820/84-364 : S-AFSEEEEEKLMAAHRLYGNKWAMIARLFPGRTDNAVKNHWHVIMARKYR
Aco_HBLgroup23g002090/12-329 : E-GFTPAEEEHIITLHAMIGSRWSIIANQLPGRTDNDIKNYWNTKLSKKLR
Aco_HBLgroup6g005640/3-277 : R-PFTPEEDETIIRAHHHHGNKWATIARLLSGRTDNAVKNHWNSTLKRKLS
Aco_HBLgroup10g001000/14-264 : G-NFTDEEDELIIKLHGLLGNRWSLIAGQLPGRTDNEIKNYWNTHIKKKLI
Aco_HBLgroup1g005280/16-303 : G-NFTPHEEGIIIHLQALLGNRWAAIASYLPQRTDNDIKNYWNTHLKKKIK
Aco_HBLgroup23g003660/14-275 : G-NISPEEQLLILELHSRWGNRWSKIAQFLPGRTDNEIKNYWRTRVQKHAK
Aco_HBLgroup11g003980/11-332 : G-SFSAQEERIIIDVHRILGNRWAQIAKHLPGRTDNEVKNFWNSCIKKKLI
Aco_HBLgroup5g009070/171-514 : D-TWSEEEDRILIEAHMEVGNKWAEIAKRLPGRTENSIKNHWNATKRRQFA
Aco_HBLgroup19g011660/64-266 : R-AFSEGEEERLLAAHQLYGNKWALIARLFPGRTDNAVKNHWHVIMARKSR
Aco_HBLgroup3g004490/53-297 : G-NFTEEEDELIIKLHSLLGNKWSLIAGRLPGRTDNEIKNYWNTHIKRKLL
Aco_HBLgroup13g010580/12-256 : G-NITQEEEDLIIRLHKLLGNRWSLIAGRLPGRTDNEIKNYWNTYLRKKVF
Aco_HBLgroup7g004270/12-219 : G-MLTEAEEWLVIHLHAKLGNRWSKIAAELSGRTDNQIKNYWNTRIKKKLV
Aco_HBLgroup7g000370/16-355 : G-RFSFEEEEAIIQLHSVLGNKWSAIAARLPGRTDNEIKNYWNTHIRKRLL
Aco_HBLgroup22g005280/1-225 : G-NITCEEEELIIRLHMLLGNRWSLIAARLPGRTDNEIKNYWNTTLAKRVD
Aco_HBLgroup8g001710/421-705 : R-NFNEDEEDLIIKLHALLGNRWSLIAGRLPGRTDDEVKSYWNSHLKKII
Aco_HBLgroup17g010700/12-258 : G-NISDDEVELIIKLHKLLGNRWSLIARRLPGRTDNEIKNYWNTNLSKKAE
Aco_HBLgroup25g003080/12-231 : G-NFTAEEEETIIRLHEMLGNRWSAIAAKLPGRTDNEIKNVWHTHLKKRLG
Aco_HBLgroup18g005320/12-290 : G-LLSESEERLVIDLHAQLGNRWSKIASHLPGRTDNEIKNHWNTHIKKKLI
Aco_HBLgroup1g005410/64-362 : R-AFSEAEEERLLTAHKKLYGNKWALIAKLFPGRTDNAVKNHWHVIMARRHR
Aco_HBLgroup17g008760/16-313 : G-KFSAEEEQTIINLHSILGNKWSAIATHLPGRTDNEIKNFWNTHLKKRLI
Aco_HBLgroup17g007040/12-212 : G-LLSESEENLVIDLHAQLGNRWSKIAARLPGRTDNEIKNHWNTHIKKKLK
Aco_HBLgroup9g002390/12-233 : G-NFTKEEEEMIFKLHELLGNRWSAIAARLPGRTDNEIKNVWHTYLKKRLM
Aco_HBLgroup11g002620/14-204 : G-NFSHDEDELIVKLHSLLATKMVIIARSLPGRTDNEIKNHWNTHIRRKLL
Aco_HBLgroup7g010980/12-334 : G-GFSEDEDRIICSLYISIGSRWSIIAAQLPGRTDNDIKNYWNTRLKKKFL
Aco_HBLgroup8g002040/20-328 : G-NLTPEEQFLILELHSKWGNRWSRIAQHLPGRTDNEIKNYWRTRVQKQAR
Aco_HBLgroup13g003150/12-316 : G-DFSDDEDRIICNLFASIGSRWSIIAAQLPGRTDNDIKNYWNTKLKKKLM
Aco_HBLgroup8g005730/12-248 : G-GFTEEEDNIIYALYQKIGSRWSVIASQLPGRTDNDIKNYWNTKLKKRMI
Aco_HBLgroup9g005260/17-360 : G-KFSEDEERLIIHLHSILGNKWSTIASRLSGRTDNEIKNYWNTHLKRKLL
Aco_HBLgroup1g003700/12-401 : G-SFSQQEENLIIELHAVLGNRWSQIAAQLPGRTDNEIKNFWNSSIKKKLR
Aco_HBLgroup16g004940/133-411 : S-AFSEEEEEKLMAAHRLYGNKWAMIARLFPGRTDNAVKNHWHVIMARKYR
Aco_HBLgroup17g010750/12-215 : G-NITQDEEDLIIRLHKLLGNRWSLIAGRIPGRTDNEIKNYWNTFLKKKVH
Aco_HBLgroup19g008140/12-418 : G-TFSEQEEKLIVELHAVLGNRWSQIAAQLPGRTDNEIKNLWNSCIKKKLR
Aco_HBLgroup22g001340/2-235 : S-PFTEEEEERLLASHRIHGNRWAVIARLFPGRTDNAVKNHWHVIMARKCR
Aco_HBLgroup25g001110/12-341 : G-DFTDDEDRIICTLFASIGSRWSIIASHLPGRTDNDIKNYWNTKLKRKLL
Aco_HBLgroup25g004340/13-355 : G-TFSLQEEGIIMKLHAMLGNRWSRIAMHLPGRTDNEVKNHWNSYLKKKVL
Aco_HBLgroup16g004760/79-411 : G-KFSIQEEQTIIQLHALLGNRWSAIATHLPKRTDNEIKNYWNTHLKKRLA
Aco_HBLgroup25g006490/14-288 : G-NFTKEEEEKLIKLHNLLGNRWSKIASFLPGRTDNEIKNIWNTHIKKKIA
Aco_HBLgroup22g004000/31-467 : G-GWSTEEDMLLCEAQKIFGNRWTEIAKVVTGRTDNAVKNRFSTLCKKRAK
Aco_HBLgroup14g001220/40-460 : G-AITQEEEDKIIQLHAMLGNKWAKMAARLPGRTDNEIKNYWNTRIKRRLR
Aco_HBLgroup21g007210/3-276 : R-PFTPDEDDAIVRAHRRFGNKWATIARLLSGRTDNAIKNHWNSTLKRKYL
Aco_HBLgroup12g005490/42-270 : K-PFTPEEDSKIIELHRKYHNGWATIARHMPGRSDNAIKNRWNCTLKNRQR
Aco_HBLgroup2g003160/39-479 : C-PFSPEEEQLVAELHAQIGNKWARMATQLTGRTDNEIKNYWNTRIKRQQR
Aco_HBLgroup9g010670/13-298 : G-MLSQAEEETVINLHAAWGNKWSQIARHLPGRTDNEVKNHWNSYLKKRVL
Aco_HBLgroup14g000300/3-257 : G-NITPEEQILILELHCRWGNRWSKIAKYLPGRTDNEIKNYWRTRVQKHAK
Aco_HBLgroup20g008650/16-347 : G-RFSEDEEKLIIHLHSVLGNKWSSIATRLPGRTDNEIKNYWNTHLRKKLL
Aco_HBLgroup22g008830/2-235 : S-PFTEEEEERLLASHRIHGNRWAVIARLFPGRTDNAVKNHWHVIMARKCR
Aco_HBLgroup19g010220/10-257 : R-PFTPAEDAAIVAAHAQFGNKWAAIARLLPGRTDNAIKNHWNSTLRRRRR
Aco_HBLgroup2g000460/60-369 : K-PFTEEEDRIIISAHAIHGNKWASIARLLEGRTDNAIKNHWNSTLRRKCF
Aco_HBLgroup9g004500/23-269 : G-NISEDEEDLIIRLHNLLGNRWSLIAGRLPGRTDNEIKNHWNTHLSKRSL
Aco_HBLgroup21g003230/25-397 : G-PFRPDEERLIIRLHALLGNKWARISSHLPGRTDNEIKNYWNTRLKRRQR
Aco_HBLgroup9g004120/16-270 : G-NFTDQEEKLIIHLQALLGNRWAAIASYLPERTDNDIKNYWNTHLKKKLM
Aco_HBLgroup25g002270/16-279 : G-KITPQEENIIIELHALWGNRWSTIARSLPGRTDNEIKNYWRTHYKKSKP
Aco_HBLgroup6g005750/12-364 : G-TFSEQEENLIIELHAVLGNRWSQIAAQLPGRTDNEIKNLWNSCLKKKLR
Aco_HBLgroup6g004420/12-272 : G-DFSPEEDKIICSLYISIGSRWSIIAAQLPGRTDNDVKNYWNTKLKKRLL
Aco_HBLgroup2g007150/12-361 : G-SFSQEEESLIIELHAILGNRWSQIAAKLPGRTDNEIKNFWNSCLKKRLR
Aco_HBLgroup23g004210/12-325 : G-NISKQEEEIIITLHATFGNRWSLIATHLPGRTDNEIKNYWNSHLSRSIH
Aco_HBLgroup16g005090/16-270 : G-NFTDQEEKLIIHLQALLGNRWAAIASYLPERTDNDIKNYWNTHLKKKLM
Aco_HBLgroup9g006470/111-416 : GCKFSAEEERVVIELQAQLGNKWARIATYLPGRTDNDVKNFWSTRQKRLAR
Aco_HBLgroup2g004610/45-277 : G-NITPQEQLLILELHSLWGNKWSKIAQHLPGRTDNEIKNYWRTRVQKHAK
Aco_HBLgroup17g010710/12-215 : G-NITQDEEDLIIRLHKLLGNRWSLIAGRIPGRTDNEIKNYWNTFLKKKVH
Aco_HBLgroup20g008660/17-347 : G-KFSKEEEKLIINLHSLLGNKWSSIAARLPGRTDNEIKNFWNTHMRKKLL
Aco_HBLgroup24g000350/13-281 : G-SFSQQEEDLIISLHGILGNRWSQIASQLPGRTDNEIKNFWNSCLKKKLV
Aco_HBLgroup17g007460/16-317 : G-KFSLQEEQTIIQLHALLGNRWSAIATHLPKRTDNEIKNYWNTHLKKRLA
Aco_HBLgroup1g005020/19-131 : D-PMSPEEAEIIVELHAILGNKWAKMVQ-----------------FIMILR

C

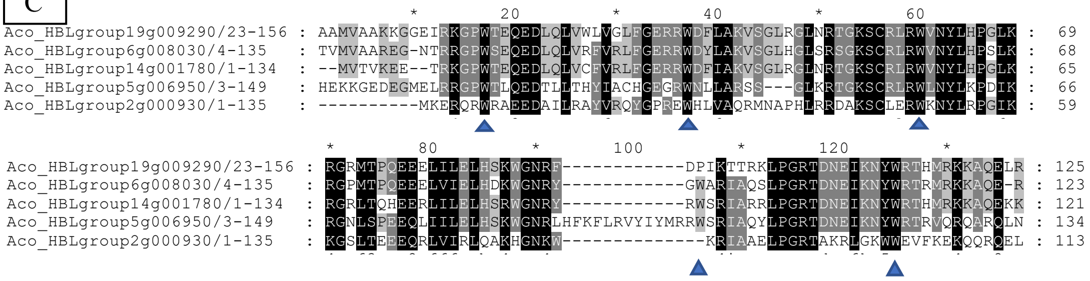

Supplementary Figure S2 Multiple sequence alignment of the AbR2R3-MYB conserved domain in *Ananas comosus* var. *bracteatus*. The shading of the alignment represents different degrees of conservation among sequences; the dark shading indicates identical residues, the light shading indicates conservative changes. The positions of the three a-helices that form each R2 (A) and R3 (B) repeat are marked as Helix 1 to Helix 3. (C), few insertions and deletions in 5 AbR2R3-MYBs, the blue triangles indicate the highly conserved tryptophan residues (W) in the MYB domain.

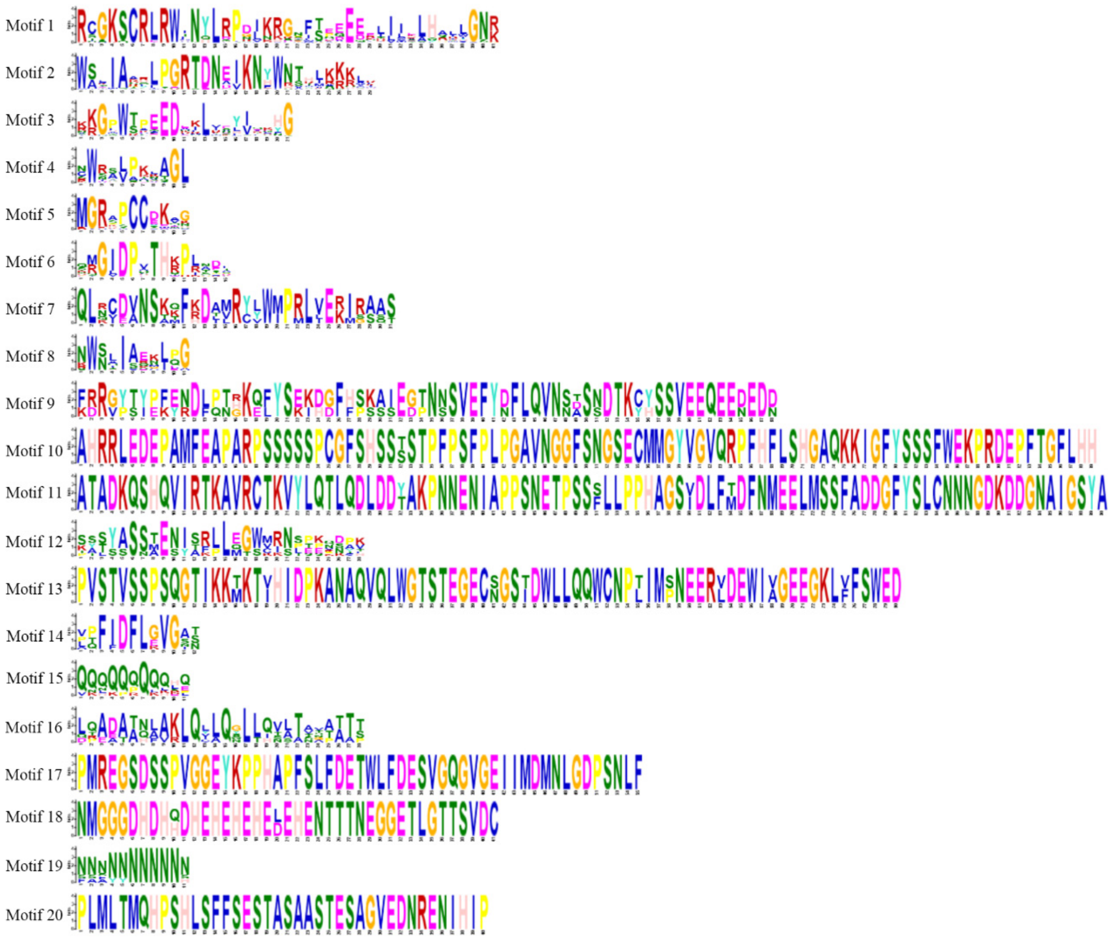

Supplementary Figure S3 A total of 20 conserved motifs were screened and visualized with different colors by using the MEME server.

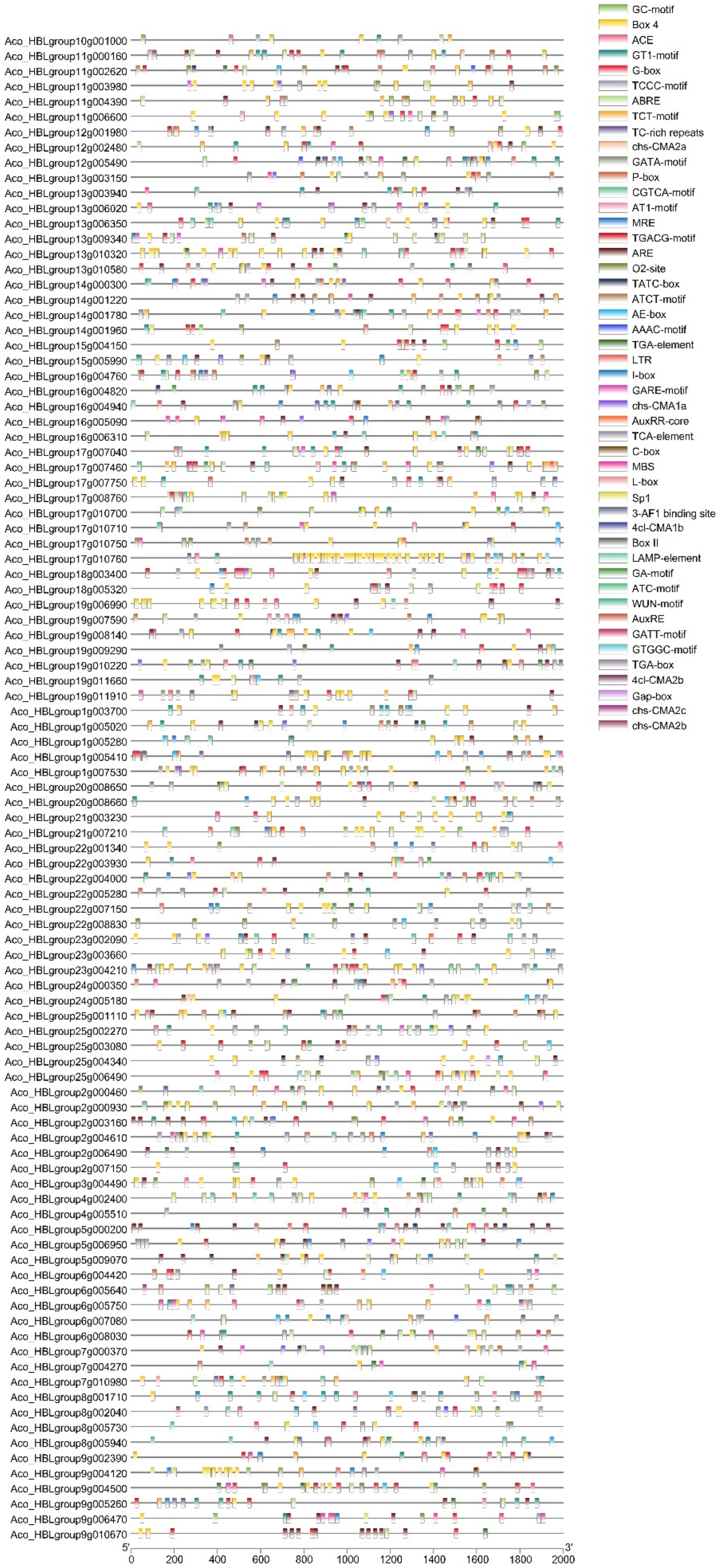

Supplementary Figure S4 Identified pivotal *cis*-elements in the promoters of AbR2R3-MYB genes in *A. comosus* var. *bracteatus*. The black line represents the upstream of the AbR2R3-MYB genes. Different colored boxes represent different *cis*-elements.

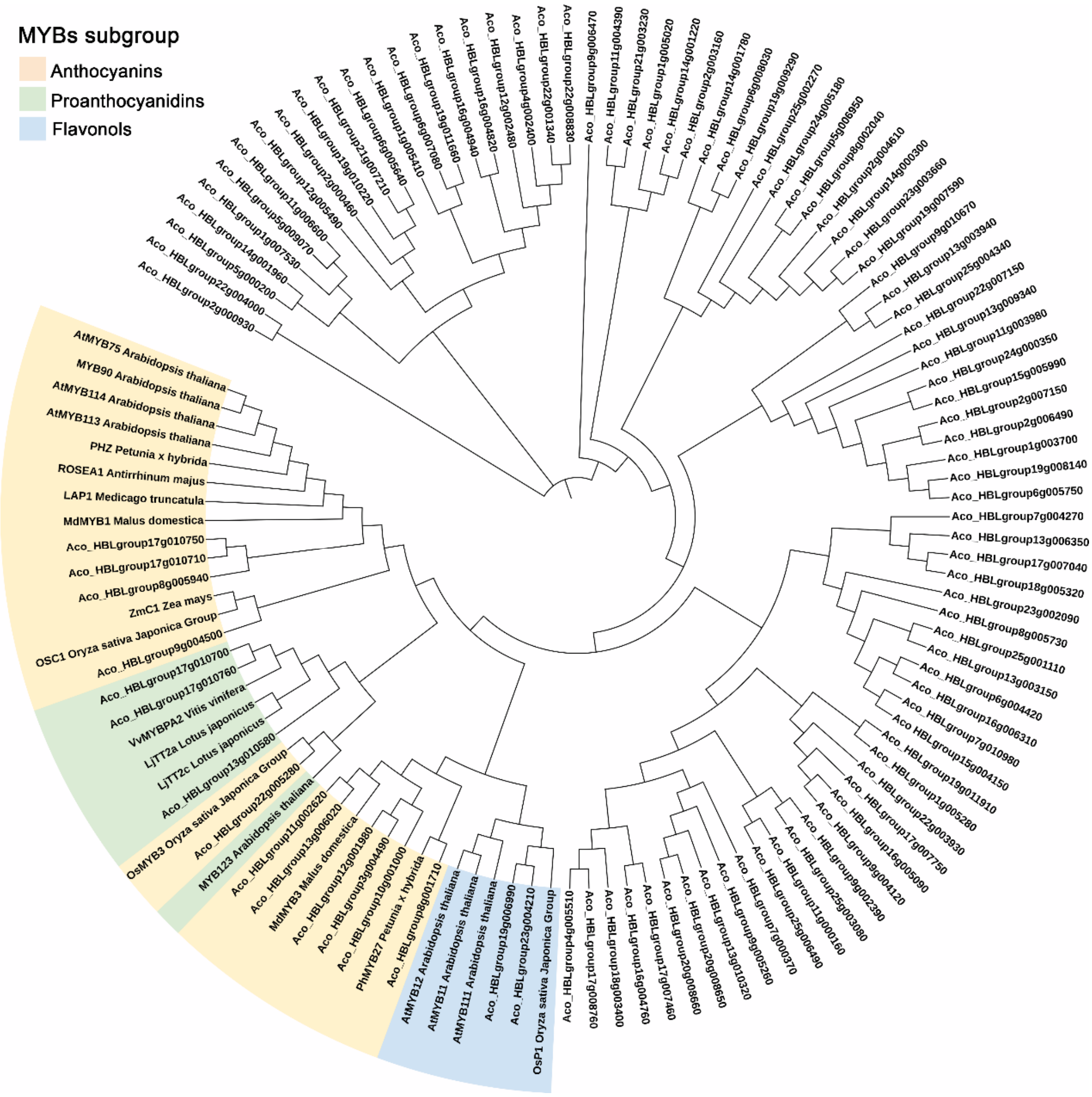

Supplementary Figure S5 Phylogenetic analysis of all AbR2R3-MYBs and functional flavonoid-related MYB proteins from other plants. The clades are shown in different colors.

Table S1. List of AbR2R3-MYB protein family genes and their physiochemical properties.

Genome ID	CDS/bp	ORF/aa	MW/kD	pI	GRAVY	Subcellular localizations
Aco_HBLgroup15g004150	708	235	26077.02	10.44	-0.536	Nucleus
Aco_HBLgroup8g001710	2115	704	76880.84	8.05	-0.266	Nucleus
Aco_HBLgroup15g005990	810	269	30218.3	5.07	-0.729	Nucleus
Aco_HBLgroup13g010580	771	256	29315	6.95	-0.748	Nucleus
Aco_HBLgroup1g005020	396	131	15032.47	9.6	-0.411	Nucleus
Aco_HBLgroup2g003160	1596	531	58719.61	4.79	-0.75	Nucleus
Aco_HBLgroup16g004760	1230	409	44995.59	8.69	-0.438	Nucleus
Aco_HBLgroup16g004820	1095	364	41056.51	6.31	-0.795	Nucleus
Aco_HBLgroup18g003400	867	288	31579.35	5.59	-0.577	Nucleus
Aco_HBLgroup16g006310	951	316	35491.63	5.39	-0.693	Nucleus
Aco_HBLgroup19g006990	1035	344	38962.26	5.29	-0.924	Nucleus
Aco_HBLgroup24g000350	846	281	31607.94	5.49	-0.656	Nucleus
Aco_HBLgroup14g001780	792	263	30568.3	8.43	-0.996	Nucleus
Aco_HBLgroup2g006490	477	158	18291.49	10.44	-0.508	Nucleus
Aco_HBLgroup23g004210	978	325	35838.72	4.89	-0.718	Nucleus
Aco_HBLgroup8g002040	987	328	36656.88	6.89	-0.63	Nucleus
Aco_HBLgroup23g002090	990	329	36305.6	6.21	-0.603	Nucleus
Aco_HBLgroup19g010220	774	257	27959.12	5.54	-0.652	Nucleus
Aco_HBLgroup11g006600	1353	450	51160.6	6.56	-0.743	Nucleus
Aco_HBLgroup22g004000	1428	475	52547.4	5.16	-0.633	Nucleus
Aco_HBLgroup8g005940	933	310	34432.3	6.95	-0.823	Nucleus
Aco_HBLgroup25g006490	867	288	32426.22	5.84	-0.85	Nucleus
Aco_HBLgroup6g005750	1095	364	40837.44	5.79	-0.678	Nucleus

Aco_HBLgroup1g007530	1287	428	48196.82	6.96	-0.794	Nucleus
Aco_HBLgroup5g006950	924	307	35109.25	7.15	-0.685	Nucleus
Aco_HBLgroup22g008830	708	235	27607.65	8.62	-1.054	Nucleus
Aco_HBLgroup13g003150	951	316	34510.72	8.59	-0.632	Nucleus
Aco_HBLgroup25g003080	696	231	26168.24	5.8	-0.694	Nucleus
Aco_HBLgroup25g004340	1068	355	39374.68	5.56	-0.534	Nucleus
Aco_HBLgroup12g001980	768	255	28294.17	8.65	-0.478	Nucleus
Aco_HBLgroup19g007590	837	278	31380.09	6.08	-0.687	Nucleus
Aco_HBLgroup1g003700	1206	401	44796.21	6.7	-0.638	Nucleus
Aco_HBLgroup2g000460	1110	369	39808.37	5.97	-0.545	Nucleus
Aco_HBLgroup7g000370	1062	353	40028.92	5.78	-0.645	Nucleus
Aco_HBLgroup12g005490	813	270	30839.69	8.87	-0.973	Nucleus
Aco_HBLgroup18g005320	873	290	32747.74	5.13	-0.742	Nucleus
Aco_HBLgroup10g001000	792	263	29573.62	8.19	-0.654	Nucleus
Aco_HBLgroup3g004490	891	296	34114.54	8.06	-0.59	Nucleus
Aco_HBLgroup22g007150	1017	338	37214.59	5.43	-0.585	Nucleus
Aco_HBLgroup7g010980	1005	334	37000	7.56	-0.508	Nucleus
Aco_HBLgroup22g005280	678	225	26073.58	8.67	-0.819	Nucleus
Aco_HBLgroup14g001960	1578	525	58691.49	8.8	-0.67	Nucleus
Aco_HBLgroup19g008140	1257	418	45961.82	5.91	-0.707	Nucleus
Aco_HBLgroup7g004270	660	219	24721.98	6.14	-0.632	Nucleus
Aco_HBLgroup14g000300	774	257	29394.99	5.76	-0.656	Nucleus
Aco_HBLgroup6g005640	834	277	29452.73	7.22	-0.538	Nucleus
Aco_HBLgroup8g005730	747	248	27722.21	6.24	-0.66	Nucleus
Aco_HBLgroup21g003230	1329	442	46707.24	6.32	-0.422	Nucleus
Aco_HBLgroup5g000200	2925	974	108600.75	5.06	-0.621	Nucleus

Aco_HBLgroup20g008660	1038	345	37119.14	8.93	-0.322	Nucleus
Aco_HBLgroup24g005180	588	195	22783.38	5.53	-0.867	Nucleus
Aco_HBLgroup9g010670	897	298	32362.82	5.94	-0.532	Nucleus
Aco_HBLgroup9g002390	702	233	26854.63	6.92	-0.617	Nucleus
Aco_HBLgroup5g009070	1545	514	57568.58	5.89	-0.628	Nucleus
Aco_HBLgroup11g002620	612	203	23283.64	8.78	-0.716	Nucleus
Aco_HBLgroup17g007750	945	314	34547.39	6.33	-0.729	Nucleus
Aco_HBLgroup23g003660	828	275	31455.08	5.18	-0.76	Nucleus
Aco_HBLgroup17g007460	948	315	34534.59	6.68	-0.628	Nucleus
Aco_HBLgroup6g004420	819	272	30524.36	7.58	-0.624	Nucleus
Aco_HBLgroup9g005260	1077	358	39318.59	6.06	-0.265	Nucleus
Aco_HBLgroup22g001340	708	235	27599.54	6.77	-1.054	Nucleus
Aco_HBLgroup19g009290	834	277	31319.75	5.46	-0.878	Nucleus
Aco_HBLgroup1g005280	906	301	33498.56	6.09	-0.621	Nucleus
Aco_HBLgroup19g011910	879	292	32761.64	5.87	-0.695	Nucleus
Aco_HBLgroup21g007210	831	276	29571.3	8.49	-0.582	Nucleus
Aco_HBLgroup9g004120	807	268	29839.42	6.54	-0.705	Nucleus
Aco_HBLgroup14g001220	1623	540	59492.37	5.28	-0.528	Nucleus
Aco_HBLgroup2g007150	1086	361	39526.02	5.38	-0.673	Nucleus
Aco_HBLgroup22g003930	849	282	31049.75	5.31	-0.479	Nucleus
Aco_HBLgroup17g010700	777	258	29364.85	7.59	-0.873	Nucleus
Aco_HBLgroup13g003940	831	276	30461.8	5.99	-0.623	Nucleus
Aco_HBLgroup12g002480	933	310	35132.69	9.23	-0.864	Nucleus
Aco_HBLgroup4g002400	687	228	26483.37	7.1	-0.982	Nucleus
Aco_HBLgroup13g006350	726	241	26973.35	7.15	-0.777	Nucleus
Aco_HBLgroup17g010750	648	215	24750.07	8.64	-0.717	Nucleus

Aco_HBLgroup19g011660	801	266	29417.98	10.22	-0.89	Nucleus
Aco_HBLgroup4g005510	552	183	21267.55	9.85	-0.62	Nucleus
Aco_HBLgroup13g010320	936	311	34610.55	9.27	-0.438	Nucleus
Aco_HBLgroup11g004390	1101	366	39443.85	5.79	-0.324	Nucleus
Aco_HBLgroup9g006470	1251	416	45743.15	6.36	-0.491	Chloroplast
Aco_HBLgroup1g005410	1089	362	39468.34	5.6	-0.688	Nucleus
Aco_HBLgroup17g007040	639	212	23571.74	7.57	-0.423	Nucleus
Aco_HBLgroup11g003980	999	332	37135.86	6.39	-0.567	Nucleus
Aco_HBLgroup17g010760	777	258	29341.87	7	-0.851	Nucleus
Aco_HBLgroup13g006020	675	224	25607.68	9.02	-0.603	Nucleus
Aco_HBLgroup25g001110	1026	341	37559.32	6.99	-0.83	Nucleus
Aco_HBLgroup17g008760	936	311	34591.95	5.83	-0.462	Nucleus
Aco_HBLgroup20g008650	1038	345	37745.54	5.99	-0.446	Nucleus
Aco_HBLgroup6g008030	720	239	27437.65	5.73	-0.843	Nucleus
Aco_HBLgroup6g007080	1047	348	38148.19	6.6	-0.708	Nucleus
Aco_HBLgroup16g005090	807	268	29853.49	6.96	-0.706	Nucleus
Aco_HBLgroup25g002270	840	279	31907.45	7.79	-1.008	Nucleus
Aco_HBLgroup9g004500	810	269	30410.06	6.51	-0.693	Nucleus
Aco_HBLgroup2g004610	834	277	31665.86	9.11	-0.402	Nucleus
Aco_HBLgroup11g000160	1077	358	39860.15	4.9	-0.825	Nucleus
Aco_HBLgroup13g009340	1038	345	38960.62	5.33	-0.634	Nucleus
Aco_HBLgroup16g004940	1236	411	45848.07	6.32	-0.631	Nucleus
Aco_HBLgroup2g000930	1056	351	40173.88	9.86	-0.865	Nucleus
Aco_HBLgroup17g010710	648	215	24674.98	8.25	-0.688	Nucleus

Table S2. Estimated Ka/Ks ratios of the duplicated R2R3-MYB genes in *Ananas comosus* var. *bracteatus*

Gene_1	Gene_2	Ka	Ks	Ka/Ks	Effective Length (bp)	AverageS-sites	AverageN-sites	Duplication Type
Aco_HBLgroup1g008320	Aco_HBLgroup14g001960	0.391894	1.022078	0.383429	1209	264.92	944.08	segmental
Aco_HBLgroup1g003700	Aco_HBLgroup19g008140	0.260106	1.962444	0.132542	1104	238.42	865.58	segmental
Aco_HBLgroup1g005280	Aco_HBLgroup19g011910	0.254155	1.583335	0.160519	834	185.33	648.67	segmental
Aco_HBLgroup1g005410	Aco_HBLgroup19g011660	0.415646	1.091040	0.380963	771	168.00	603.00	segmental
Aco_HBLgroup10g001000	Aco_HBLgroup12g001980	0.216943	1.764469	0.122951	702	166.75	535.25	segmental
Aco_HBLgroup10g001000	Aco_HBLgroup3g004490	0.229569	2.676690	0.085766	762	181.33	580.67	segmental
Aco_HBLgroup13g006350	Aco_HBLgroup7g004270	0.338766	0.698805	0.484778	621	141.33	479.67	segmental
Aco_HBLgroup13g003940	Aco_HBLgroup9g010670	0.491340	3.734796	0.131557	795	185.83	609.17	segmental
Aco_HBLgroup14g000300	Aco_HBLgroup19g007590	0.241417	1.837748	0.131366	747	172.92	574.08	segmental
Aco_HBLgroup15g004150	Aco_HBLgroup8g005730	0.414147	1.624802	0.254891	633	149.08	483.92	segmental
Aco_HBLgroup16g004820	Aco_HBLgroup16g004940	0.005894	0.004335	1.359532	1083	231.33	851.67	tandem
Aco_HBLgroup16g004760	Aco_HBLgroup17g007460	0.171706	1.243373	0.138097	906	209.33	696.67	segmental
Aco_HBLgroup16g005090	Aco_HBLgroup17g007750	0.201515	1.018050	0.197942	786	179.08	606.92	segmental
Aco_HBLgroup16g005090	Aco_HBLgroup22g003930	0.340588	1.993741	0.170829	762	173.25	588.75	segmental
Aco_HBLgroup16g005090	Aco_HBLgroup9g004120	0.001608	0.016714	0.096217	804	181.50	622.50	segmental
Aco_HBLgroup17g007040	Aco_HBLgroup18g005320	0.146895	0.646526	0.227206	609	139.25	469.75	segmental
Aco_HBLgroup17g008760	Aco_HBLgroup18g003400	0.198171	1.001056	0.197962	765	171.17	593.83	segmental
Aco_HBLgroup17g007750	Aco_HBLgroup22g003930	0.244771	2.235645	0.109486	753	172.42	580.58	segmental
Aco_HBLgroup17g010700	Aco_HBLgroup22g005280	0.462982	1.698785	0.272537	591	137.25	453.75	segmental
Aco_HBLgroup17g008760	Aco_HBLgroup4g005510	0.160713	1.206373	0.133220	534	118.08	415.92	segmental
Aco_HBLgroup17g007040	Aco_HBLgroup4g001840	0.340427	1.549228	0.219740	450	101.92	348.08	segmental
Aco_HBLgroup18g005320	Aco_HBLgroup4g009920	0.356364	2.427242	0.146818	639	141.33	497.67	segmental

Aco_HBLgroup18g003400	Aco_HBLgroup4g005510	0.232726	1.534717	0.151641	531	118.67	412.33	segmental
Aco_HBLgroup18g005320	Aco_HBLgroup4g001840	0.233265	2.022556	0.115332	453	102.58	350.42	segmental
Aco_HBLgroup19g007590	Aco_HBLgroup2g004610	0.276877	1.454797	0.190320	726	170.25	555.75	segmental
Aco_HBLgroup19g006990	Aco_HBLgroup23g004210	0.309376	1.443415	0.214336	861	177.58	683.42	segmental
Aco_HBLgroup19g007590	Aco_HBLgroup23g003660	0.200221	1.314296	0.152341	795	178.50	616.50	segmental
Aco_HBLgroup2g006490	Aco_HBLgroup2g007150	0.039619	0.153185	0.258634	381	83.00	298.00	tandem
Aco_HBLgroup22g001340	Aco_HBLgroup22g008830	0.010946	0.019891	0.550297	705	152.83	552.17	tandem
Aco_HBLgroup22g001340	Aco_HBLgroup4g002400	0.156896	0.988617	0.158703	639	138.67	500.33	segmental
Aco_HBLgroup22g008830	Aco_HBLgroup4g002400	0.146106	1.077750	0.135565	639	139.33	499.67	segmental
Aco_HBLgroup22g001340	Aco_HBLgroup4g009300	0.228640	1.272632	0.179659	543	115.50	427.50	segmental
Aco_HBLgroup25g003080	Aco_HBLgroup9g002390	0.262922	1.418641	0.185334	675	150.08	524.92	segmental
Aco_HBLgroup4g002400	Aco_HBLgroup4g009300	0.011228	0.024525	0.457802	573	124.33	448.67	tandem
Aco_HBLgroup5g000200	Aco_HBLgroup8g007840	0.313663	1.117426	0.280701	2469	548.67	1920.33	segmental
Aco_HBLgroup5g006950	Aco_HBLgroup8g002040	0.302289	2.064544	0.146419	825	186.92	638.08	segmental

Table S3. Functionally annotated *cis*-elements identified in the promoters of AbR2R3-MYB genes in *Ananas comosus* var. *bracteatus*

**Categories**	***Cis*-Elements**	**Functions of *Cis*-Elements**
Light-response elements	Box 4, ATCT-motif, ATC-motif	part of a conserved DNA module involved in light responsiveness
	ACE	cis-acting element involved in light responsiveness
	GT1-motif, AAAC-motif, Sp1, 3-AF1 binding site, 4cl-CMA1b, 4cl-CMA2b	light responsive element
	G-Box, C-box	cis-acting regulatory element involved in light responsiveness
	TCCC-motif, TCT-motif, chs-CMA2a, GATA-motif, AT1-motif, I-box, chs-CMA1a, L-box, Box II, LAMP-element, GA-motif, GATT-motif, GTGGC-motif, chs-CMA2c, chs-CMA2b, Gap-box	part of a light responsive element
	MRE	MYB binding site involved in light responsiveness
	AE-box	part of a module for light response
Stress-response elements	GC-motif	enhancer-like element involved in anoxic specific inducibility
	TC-rich repeats	cis-acting element involved in defense and stress responsiveness

	ARE	cis-acting regulatory element essential for the anaerobic induction
	LTR	cis-acting element involved in low-temperature responsiveness
	MBS	MYB binding site involved in drought-inducibility
	WUN-motif	wound-responsive element
Hormone-response elements	ABRE	cis-acting element involved in the abscisic acid responsiveness
	P-box, GARE-motif	gibberellin-responsive element
	TATC-box	cis-acting element involved in gibberellin-responsiveness
	CGTCA-motif, TGACG-motif	cis-acting regulatory element involved in the MeJA-responsiveness
	O2-site	cis-acting regulatory element involved in zein metabolism regulation
	TGA-element	auxin-responsive element
	AuxRR-core	cis-acting regulatory element involved in auxin responsiveness
	AuxRE, TGA-box	part of an auxin-responsive element

	TCA-element	cis-acting element involved in salicylic acid responsiveness

Table S4. List of primers used for qRT-PCR.

Gene Name	Primer Sequences (5'-3')	
Unigene.16454	Forward: TCTCACGCCCTCTTTCTTCCA	Reverse: GCTCTAACTCGCCACGCCTTT
Unigene.16459	Forward: GGATTTGAAGCATCATGGCACA	Reverse: AGCACAACCCACCTCATTTCG
SDP	Forward: TGGGCGGCGTATTTACTGTGG	Reverse: CCAAACCTATCCTTCGCCATC
IDH	Forward: GAGTCTATTCGGGCCTTTGCT	Reverse: CCCAGACATACCCTCCCTCAC
Aco_HBLgroup8g001710	Forward: CTAAGCAACGCCCCGCAGTATG	Reverse: TGGCAGGAAGAATGACGCTATCG
Aco_HBLgroup3g004490	Forward: CTCTCCCCAAAGCCGCAGG	Reverse: CGACCAGCAATCAAGGACCAT
Aco_HBLgroup10g001000	Forward: CCTCTCAATAATAAGCCTCCCG	Reverse: CCAAGGTGGTAGCACAAGC
Aco_HBLgroup8g005940	Forward: CCCTACTCCTGCTCCCGCTGAAG	Reverse: GCTGGTGCTGCTGTAGGAAGATG
Aco_HBLgroup22g005280	Forward: TCACAAGAAGCACCAAGAACTCCTC	Reverse: ACTCCAACTTGCCGCGAACTTAG
Aco_HBLgroup13g006020	Forward: CCTCCTCGGCAACAAATGGTCTC	Reverse: CCTCCTTATGTGCGTGTTCCAGTAG
Aco_HBLgroup12g001980	Forward: GGACGAAGGAGGAGGACCAGAG	Reverse: GAAGAAGACCTGCGGCTTTGGG
Aco_HBLgroup17g010710	Forward: TTTGATAGCGGGAAGGATACC	Reverse: CTTGAGATGGGCTTGACACG
Aco_HBLgroup17g010750	Forward: GAACCCACCCTTGCTCTCA	Reverse: CGGTATCCTCCCCGCTATC
Aco_HBLgroup11g002620	Forward: ACCACTGGAACACGCACATCA	Reverse: CAAAAGAAGCGGCGAAATC
